# *Pongamia pinnata* (L.) Pierre leaf extract: *in vitro*, *in vivo*, and *in silico* approaches to combat the waterborne bacterial pathogens associated with diarrheal conditions

**DOI:** 10.3389/fcimb.2026.1830625

**Published:** 2026-07-20

**Authors:** Awadhesh Kumar, T. B. C. Laldingliani, Rakesh Kumar, Nurpen Meitei Thangjam, Ramdinmawia Vuite, Sumanta Kumar Sahu, Ravi Rawat, Tapan Behl, Volkan Eyupoglu

**Affiliations:** 1 Department of Botany, Mizoram University, Aizawl, India; 2 Department of Bioinformatics, Central University of South Bihar, Gaya, Bihar, India; 3 Department of Pharmacy, Saraswati Institute of Management and Para Medical Sciences, Imphal, India; 4 Department of Zoology, Mizoram University, Aizawl, India; 5 Amity Institute of Pharmacy, Amity University, Noida, Uttar Pradesh, India; 6 Department of Chemistry, Cankiri Karatekin University, Cankiri, Türkiye

**Keywords:** antioxidant, diarrheal disease, GC-MS, molecular docking, molecular dynamics

## Abstract

*Pongamia pinnata* (L.) Pierre, a medicinal plant traditionally used in Indian ethnomedicine, was evaluated for its antidiarrheal, antibacterial, and antioxidant properties through a combination of *in vitro*, *in vivo*, and *in silico* approaches. This study aims to study the traditional use of plants in medicine and identify a suitable secondary metabolite that can help reduce bacterial growth in living systems, thereby lowering the risk of waterborne diseases and diarrhea. Screening of *P. pinnata* leaf extract confirmed the presence of various bioactive classes, including alkaloids, flavonoids, etc., and that it also had strong antioxidant activity. *In vitro* antibacterial assays revealed that the plant extract effectively inhibited multiple waterborne pathogens, particularly *Shigella dysenteriae* Castellani and Chalmers, 1919, and *in vivo* studies also showed significant dose-dependent antidiarrheal effects. Acute oral toxicity tests at high doses confirmed the extract’s safety, with no adverse effects on body weight, behavior, or vital organs. GC–MS analysis revealed various secondary metabolites with N-decanoic acid as the most abundant in the plant extract. Notably, 1,2,5,6-di-O-isopropylidene-3-O-methanesulfonyl glucofuranose and spiro{2,4}heptane derivatives exhibited stronger binding affinities than the standard drug ampicillin in molecular docking studies with penicillin-binding proteins (PBPs). These findings suggest that *P. pinnata* harbors potent metabolites with significant antidiarrheal and antibacterial activity, offering promise for the development of plant-based therapeutics against waterborne bacterial pathogens. The integration of experimental and computational approaches strengthens its candidacy for future drug development targeting diarrheal diseases.

## Introduction

Diarrheal diseases are a common bacterial infection affecting people worldwide, accounting for 3.2% of all deaths according to WHO (2004). It is the second largest cause of years of productive life lost due to premature mortality and disability (Bayode et al., 2026). Despite economic and healthcare progress, diarrhea is projected to remain a leading health problem by 2020 (Murray and Lopez, 1997a and b), mainly due to lack of access to clean water in many developing countries. Contaminated water with bacteria such as *Escherichia coli* Escherich 1885, *Vibrio cholerae* Pacini 1854, *Salmonella enterica serovar* Typhi Browicz 1874, and others can cause diseases such as cholera, dysentery, typhoid, and gastrointestinal symptoms (Kumar et al., 2013). This situation creates opportunities for cost-effective approaches, with medicinal plants potentially filling this gap. Thus, plants produce bioactive molecules that serve as natural medicines vital to drug development (Baker et al., 1995). India’s leprosy biodiversity encompasses species, genetic diversity, habitat variety, and various agro-climatic conditions (Bhattacharjee, 1998; Zafar et al., 1999). It also utilizes different plants and parts in traditional medicine for treating ailments (Laldingliani, 2022).

*P. pinnata*, also known as Karanja in Hindi, Indian beech in English, and Pongam in Tamil (Krishnamurthi, 1969), is a plant belonging to the family Fabaceae. The plant is a monotypic genus and grows abundantly throughout India and Myanmar and is also distributed from tropical Asia to Northern Australia. It is an evergreen medium-sized tree that grows primarily in seasonally dry tropical biomes, with fast-growing, shiny green, compound leaves and fragrant white-to-purple pea-like flowers. It is valued for producing seed oil, providing shade, and enduring drought.

This plant offers multipurpose benefits and potential as a biodiesel source (Naik et al., 2008). In traditional medicine, different parts treat diseases like bronchitis, cough, rheumatism, and diabetes (Kirtikar and Basu, 1995). Leaf juice/decoction fights colds, coughs, diarrhea, dyspepsia, flatulence, gonorrhea, and leprosy and acts as an antiseptic. Oil is used as an ointment for rheumatism (Shoba and Thomas, 2001; Punitha et al., 2006). Dried leaves repel insects during grain storage. The leaves have digestive, laxative, and anthelmintic properties and treat piles, wounds, and inflammations (Chopade, 2008). The roots clean gums; the leaves are used as green manure to reduce nematodes. The twigs are used as chew sticks for cleaning teeth. The bark is used internally for the treatment of bleeding piles.

Plant metabolites, including alkaloids, phenols, steroids, carbohydrates, glycosides, proteins, saponins, flavonoids, tannins, gums, and mucilages, were reported (Birajdar et al., 2011). Pharmacological bioactivities such as anti-inflammatory, anti-plasmodial, antioxidant, anti-hyperammonemic, anti-diarrheal, anti-ulcer, anti-hyperglycemic, and anti-lipid peroxidative activities were reported (Chopade, 2008).

This study on *P. pinnata* is comparable to the work on *Viola canescens* Wall. by Ahmad et al., 2023 as both employed integrated *in vivo* and *in silico* approaches to validate traditional antidiarrheal uses. However, the study mainly focused on antidiarrheal activity through castor-oil-induced diarrhea, charcoal meal assays, and opioid-receptor-targeted molecular docking to explain intestinal motility inhibition.

In contrast, the present study adopts a broader approach by evaluating antidiarrheal, antibacterial, antioxidant, and toxicity activities using integrated *in vitro*, *in vivo*, and *in silico* methods. Unlike the earlier study, which targeted diarrhea-related physiological pathways, the present work specifically focuses on waterborne diarrheagenic pathogens, particularly *S. dysenteriae*. Moreover, molecular docking targets penicillin-binding proteins (PBPs), which are associated with bacterial cell wall synthesis and antimicrobial resistance, providing a novel approach (Lawani et al., 2024). This comprehensive strategy will not only verify the ethnomedicinal use of *P. pinnata* but also provide new insights into plant-based therapeutics for waterborne gastrointestinal infections and diarrheal diseases.

## Materials and methods

### Plant materials and secondary metabolite screening

*P. pinnata* leaves were collected from Teliarganj, Prayagraj, Uttar Pradesh, India. The leaf part was used, and a methanolic extract was prepared using a Soxhlet apparatus. The resulting light green extract was filtered; the solvent was evaporated using a vacuum rotatory evaporator, and the crude extract was carefully obtained to preserve plant metabolites. The crude extract was stored in sealed vials at 4°C for further analysis.

The plant metabolite analysis was conducted on the powdered extract using established methods as preferred by Malsawmdawngliana (2021) and Ralte et al. (2022) to identify the different classes of metabolites present in this plant.

### Antioxidant activities

The antioxidant activities of the methanol extract from *P. pinnata* leaves were assessed through various methods, such as DPPH and superoxide free radical scavenging activity, and its reducing power using the previous methods mentioned by Thangjam et al. (2020). These assays provided insights into the plant’s antioxidant capabilities, suggesting its potential health benefits.

### *In vitro* antibacterial investigation

Briefly, a study on the antibacterial properties of an extract utilized the disc diffusion method for screening, as previously mentioned by Kumar et al. (2012). Bacterial cultures such as *E. coli, S. dysenteriae, V. cholerae, Salmonella enterica* serovar Typhimurium Smith 1884, and Klebsiella pneumoniae Trevisan 1887 were prepared using a 0.5 McFarland standard and spread onto Mueller–Hinton agar. Paper discs soaked in different concentrations of methanolic extract were placed on the bacterial lawn. Inhibition zones were measured with ampicillin and DMSO as controls. MIC was determined using broth microdilution ( NCCLS, 2003), and growth inhibition was evaluated by absorbance. MIC was the lowest concentration that prevented visible bacterial growth, while MBC was the concentration at which replicates failed to grow even after reinoculation. The study followed specific protocols and controls for accurate assessment.

### *In vivo* investigation

#### Experimental animal

Adult female Swiss albino mice weighing between 20 and 30 g were used in this study. The animals were housed in well-ventilated polypropylene cages under controlled environmental conditions (25 °C ± 2 °C), with a 12-h light/dark cycle and free access to standard food and water. All experimental protocols were carried out in accordance with the guidelines approved by the Institutional Animal Ethics Committee of Mizoram University (approval no. MZU/IAEC/2021-22/06).

#### Acute oral toxicity test

At first, an acute oral toxicity test was performed following OECD Guideline (OECD, 2015) 425 using five female Swiss albino mice. A single oral limit dose of 2,000 mg/kg body weight was administered. Initially, one mouse received the dose and was observed for 24 h. Upon survival, the remaining four mice were sequentially dosed at the same level, bringing the total to five animals. Each mouse was monitored individually for signs of toxicity at least once within the first 30 min post-administration and then periodically throughout the first 24 h and daily for the following 14 days, thus completing the observation period. The parameters, including mortality, respiration, sedation, body posture, diarrhea, drowsiness, skin coloration, fur condition, and loss of consciousness (coma), were carefully monitored throughout the study. The body weight of the mice was recorded weekly from day one up to day 14. At the end of the 14-day observation period, the animals were sacrificed, and the major vital organs were excised, weighed, and examined for morphological changes.

#### Castor-oil-induced diarrhea in mice

The experiment was conducted following the method described by Awouters et al., 1978. A total of 30 mice were fasted for 18 h and randomly assigned to five groups, each consisting of six animals. Group I received distilled water (10 mL/kg b.w.) and served as the negative control, while group II received loperamide (3 mg/kg b.w.) as the positive control. Groups III, IV, and V were administered the test extract at doses of 200, 400, and 600 mg/kg b.w., respectively. At 1 h after treatment, all animals were orally administered 0.5 mL of castor oil to induce diarrhea. The mice were then placed individually in separate cages, and the severity of diarrhea was monitored over 6 h. The total number of fecal outputs, both dry and wet (diarrheal), was recorded for each animal and compared to the negative control group. After observation, the animals were sacrificed, and the major vital organs were excised and examined for morphological changes, while the colon was used for pathological examination.

The diarrheal score of the negative control group was considered 100%, and the percentage inhibition of total defecation and diarrhea was calculated using the following formulas


$$\%\;\text{of Inhibition}=\frac{\text{Mean defecation }(\text{Control}-\text{Test})}{\text{Mean defecation of Control}}\times 100$$

### GC–MS analysis

A GC–MS instrument was employed to analyze the bioactive metabolites present in the leaves. It consisted of a Clarus 690 Perkin/Elmer (Auto system XL) Gas Chromatograph, which was coupled with a mass detector, a turbo mass gold Perkin Elmer Turbomass 5.1 spectrometer, and an Elite 1 (100% dimethyl poly siloxane) capillary column measuring 123.5 M × 678 M. The instrument was initially set to 40 °C, then ramped to 115 °C at 5 °C/min, and held at 115 °C for 5 min. The temperature was then ramped to 140 °C at 5 °C/min and held for 5 min, followed by a ramp to 210 °C at 2 °C/min and a hold for 8 min. The temperature was maintained at 210 °C for 3 min, after which the oven temperature was increased to 250 °C at 5 °C/min and maintained for 9 min. The injection port temperature was set at 250 °C, while the helium flow rate was maintained at 1.5 mL/min. The ionization voltage used was 70 eV, and the samples were injected in a 10:1 split mode. The mass spectral scanning range was set to 500–800 (m/z), and the ion source temperature and interface temperature were kept at 230 °C and 240 °C, respectively. The MS start time was 3 min, the end time was 30 min, and the solvent cut time was 3 min. The spectra of volatile compounds detected using GC–MS were compared and matched with the NIST17 online library ver. 2.339.

### *In silico* study of characterized molecules

#### Protein and ligand structure preparation

The molecular structures of the bioactive compounds character*ized* by GC–MS (**Table 3**) were downloaded from the PubChem database to investigate their potential as inhibitors of membrane-bound penicillin-binding proteins (PBPs), which serve as receptor proteins. The 3D structures of the ligand molecules were downloaded in SDF format and then converted to PDBQT format using the Open Babel tool on Linux version 16.04. The 3D structures of PBPs were downloaded from the PDB database (https://www.rcsb.org), and the protein structures were pre-processed by removing water molecules and adding polar hydrogen atoms. After that, protein–ligand interactions were calculated using AutoDock 4.2.

#### *In silico* ADME test to evaluate the drug-likeness properties of molecules

An absorption, distribution, metabolism, and excretion (ADME) test was performed to evaluate the drug likeness of bioactive compounds using the online Swiss ADME tool (https://www.swissadme.ch). Several pharmacokinetic parameters were assessed to investigate the bioactive compounds of P. pinnata. For the ADME analysis, the SMILES strings for each drug were obtained from the PubChem database. There are several parameters under ADME testing that molecules must pass, such as physicochemical parameters, lipophilicity, water solubility, pharmacokinetics, drug likeness, and medicinal chemistry (Daina et al., 2017). This work mostly considers molecules based on Lipinski’s Rule of Five (2001). In particular, physicochemical parameters indicative of drug likeness, such as molecular refractivity (MR) and polar surface area (PSA), were calculated for further analysis.

#### Identification of receptor protein and molecular docking of bioactive molecules of P. pinnata

Protein target genes against ampicillin were obtained from the Swiss Target Prediction database (http://www.swisstargetprediction.ch/). Docking was performed using AutoDock Vina. The docking of ligand molecules (bioactive compounds) was performed on a defined grid (active site) of the receptor protein. For docking, the x, y, and z coordinates were set to 26.199, 50.872, and 48.246, respectively, whereas a 40 × 40 × 40 grid was used. The docking of ligands was established in terms of binding score (Gibbs free energy—ΔG), a lesser value of ΔG indicates better binding of ligands with the receptor protein. Then, the protein–ligand complexes were used for molecular interaction analysis, which was carried out using structure visualization tools, i.e., Pymol and Chimaera. Later, hydrophobic interactions were analyzed with LigPlus software.

### Protein–protein interaction and functional enrichment analysis

A protein–protein interaction (PPI) network for P. pinnata against diarrhea was constructed using the STRING database (http://string.db.org) to investigate functional relationships between proteins. The network was retrieved and analyzed in Cytoscape 3.9.1 to identify hub genes, and for the functional annotation, we used the David Bioinformatics tool (https://davidbioinformatics.nih.gov/).

### Molecular dynamics simulation study to evaluate the binding stability of ligand molecules

The protein–ligand structure stability was confirmed through MD simulations using GROMACS 2022 tool. All protein–ligand systems for MD simulations were built in cubic periodic boundary conditions (PBC) under CHARMM36 all-atom force field (July 2022) using GROMACS 2022 platforms (López et al., 2022). The protein–ligand complexes in the PBC box were placed at the center, and systems were solvated with an explicit TIP3P water model at a 1-nm marginal radius from the surface of the protein. The systems were neutral*ized* by adding the four Na+ ions. To remove the bad contacts, energy minimization was performed using the steepest descent method. The optim*ized* protein–ligand systems were further used for NVT (Berendsen et al., 1995) ensemble, where the systems were gradually heated up to 300 K for 10,000 ps, and in NPT (Berendsen et al., 1984) ensemble the systems were equilibrated at 300 K, and 1 atm pressure for 10,000 ps was applied using a leap-frog integrator. The final production run for all protein–ligand complex simulated systems was performed over 100 ns. The simulation ran for 2 fs and the coordinates were stored every 20 ps. Analysis of the resulting simulated trajectories was carried out in terms of root mean square deviation (RMSD), root mean square fluctuation (RMSF), radius of gyration (Rg), solvent accessibility surface area (SASA), principal component analysis (PCA), and hydrogen (H)-bond using GROMACS tools, and the results were visualized using GRACE tool (Kumar et al., 2022).

### Statistical analysis

All statistical analyses were accomplished by Pearson correlation using R software (version 3.4.0) to check the relationship between the variables. Results were expressed in terms of mean ± SEM, and a correlation value of P < 0.05 was considered statistically significant.

## Results

The screening of secondary metabolites of the methanolic extract of *P. pinnata* leaves revealed that different classes of bioactive compounds were present, in which proteins, amino acids, reducing sugars, and anthraquinones were absent. Based on the presence or absence of color changes, the obtained results are summar*ized* in **Table 1**.

**Table 1:** Identified class of metabolites in the methanolic extract of *P. pinnata* leaves.

Sl. no.	Test	Present/absent	Sl. No.	Test	Present/absent
1	Alkaloids	**+**	7	Terpenoids	**+**
2	Flavonoids	**+**	8	Fixed oil and fats	**+**
3	Cardiac Glycosides	**+**	9	Reducing Sugar	**-**
4	Saponins	**+**	10	Amino acids	**-**
5	Steroids	**+**	11	Proteins	**-**
6	Tannins	**+**	12	Anthraqui nones	**-**

+, presence; –, absence.

### Antioxidant activity of *P. pinnata* plant extract

**Figure 1:**
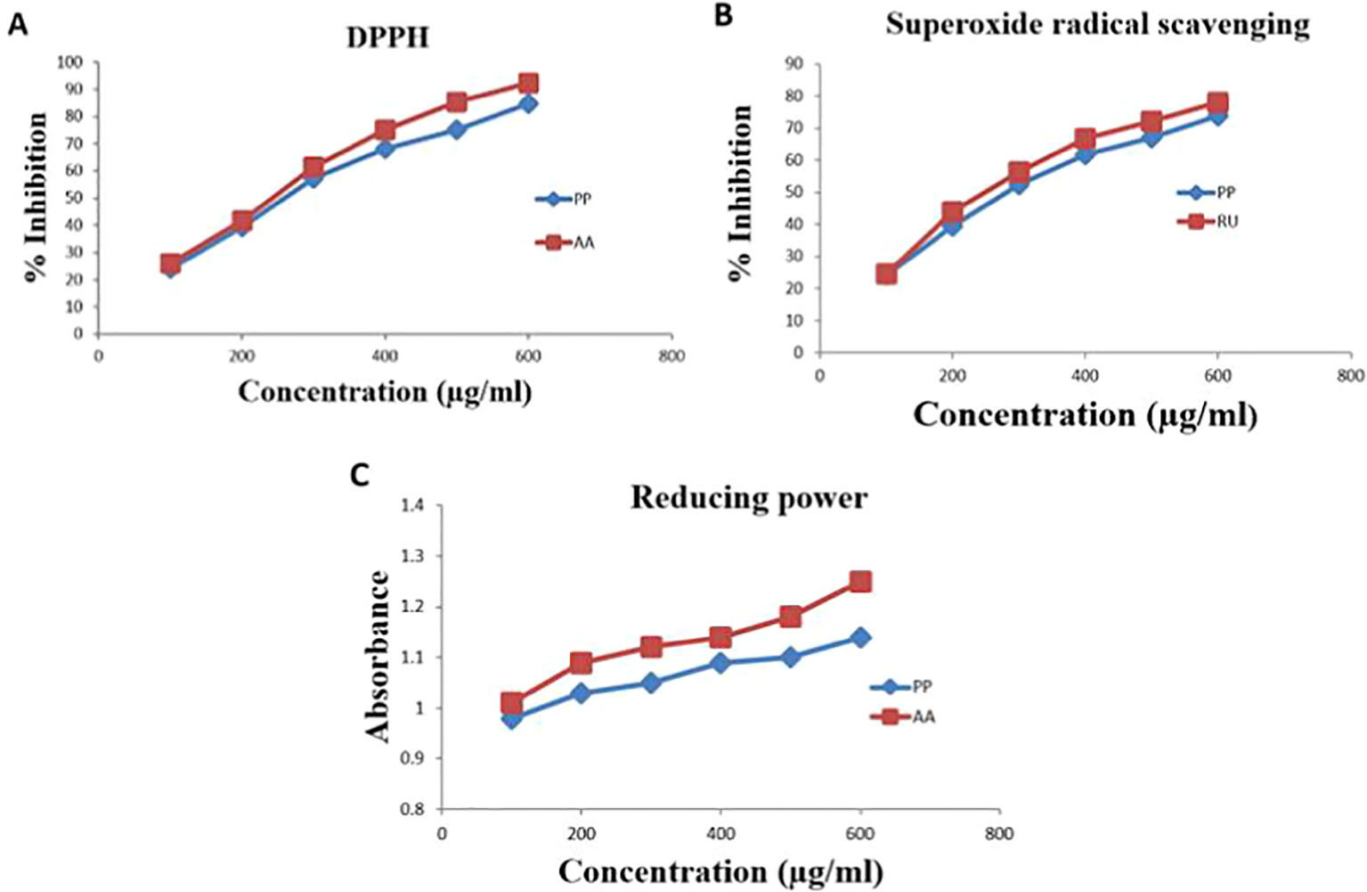
**(A)** DPPH radical scavenging activity. **(B)** Superoxide radical scavenging activity. **(C)** Reducing power.

In our current investigation, the leaf methanolic extract from this plant showed robust scavenging activity against the DPPH radical, which is attributed to its strong hydrogen-donating capacity. This scavenging property exhibited a concentration-dependent pattern. The IC_50_ values for the plant extract and standard ascorbic acid were 281.71 and 249.88, respectively. The plant extract exhibited stronger inhibitory effects than the benchmark ascorbic acid, as shown in **Figure 1A**.

The assessment of superoxide radical scavenging also exhibited a notably effective scavenging ability (**Figure 1B**). The extract’s inhibitory effects, IC50, were recorded as 317.59, while the standard rutin IC_50_ was found to be 253.25.2. This trend indicates that higher concentrations of the extract correlate with greater inhibition of the radical, thereby indicating a heightened antioxidant potential. The increasing concentration of the methanolic extract increased its reducing power (**Figure 1C**). This enhancement is attributed to the reduction of ferric ions to ferrous ions by the extract’s reducing agents.

### Antibacterial activities of *P. pinnata* plant leaf extract

**Figure 2:**
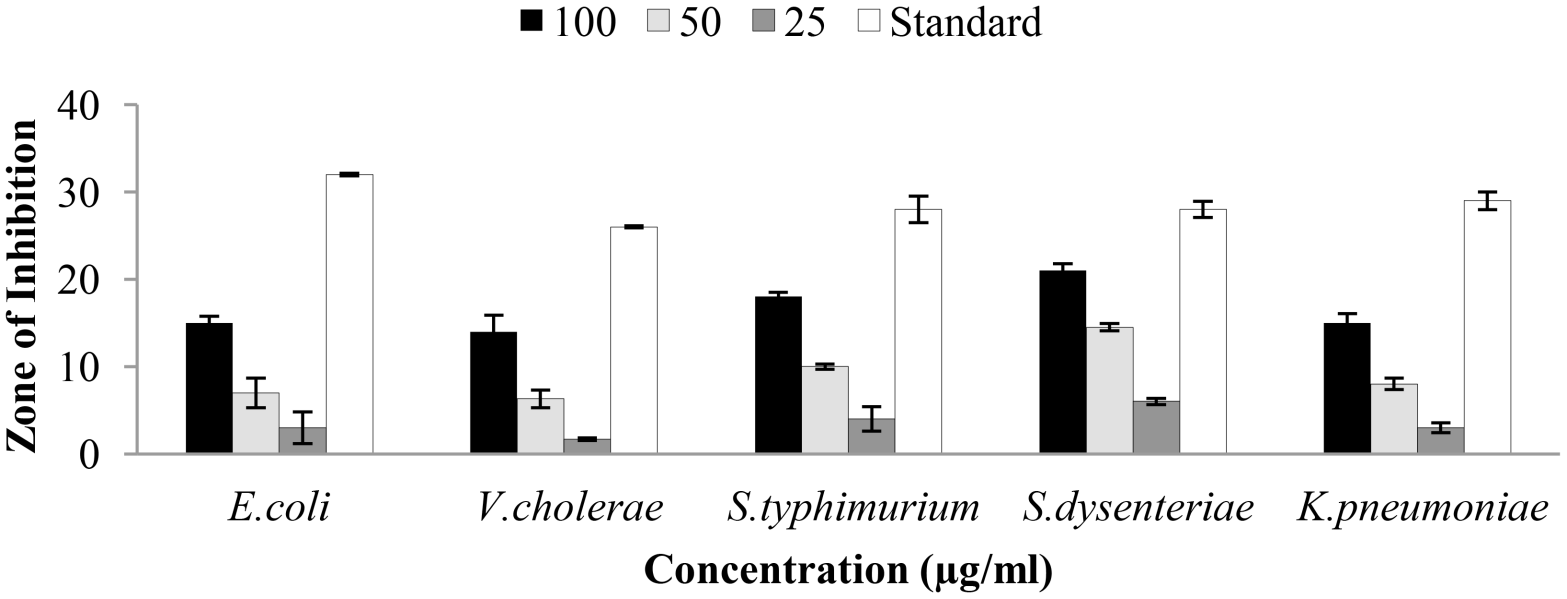
Zone of inhibition against bacterial pathogens.

The leaf methanolic extract demonstrated varying degrees of inhibitory activity against a range of human pathogenic bacteria at distinct concentrations. Notably, the highest concentration (100 mg/mL) showed the largest zone of inhibition, followed by 50 and 25 mg/mL. Particularly, the significant zone of inhibition was observed against *Shigella dysenteriae* (21 ± 0.76, 14.5 ± 0.42, and 6 ± 0.34), while the lowest inhibition zone was noted for *Vibrio cholerae* (14 ± 1.9, 6.3 ± 1.01, and 1.7 ± 0.15). Standard ampicillin showed the largest inhibition zone against *Escherichia coli* (32 ± 0.14), whereas the negative control exhibited no bacterial inhibition. The comprehensive findings from the disc diffusion technique, expressed as inhibition zones, are shown in **Figure 2**.

**Figure 3:**
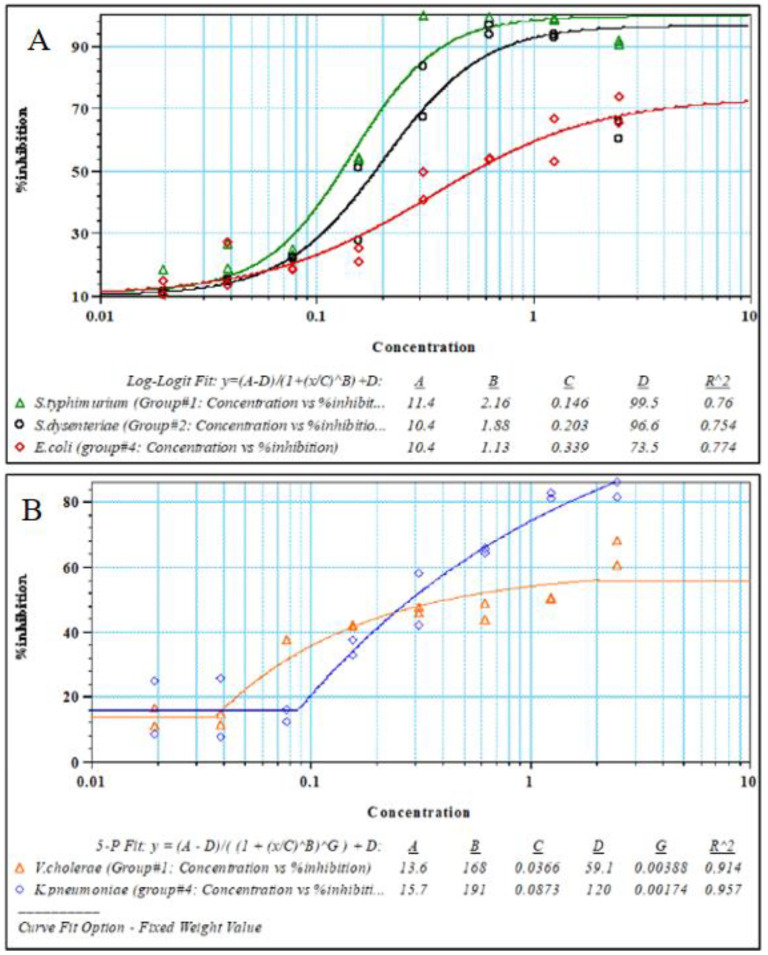
**(A)** Average percent growth inhibition. **(B)** Average percent growth inhibition.

MIC and MBC of the extract spanning from 0.625 to 5 mg/mL, respectively, were observed against the range of pathogens showing static toxicity (as illustrated in **Table 2**). Generally, the higher the MIC and MBC values, the lower the antibacterial activity. The plant extract showed the greatest efficacy against *S. dysenteriae*, while it exhibited moderate efficacy against *V. cholerae*. The visual representation of the average percentage of growth inhibition is shown in **Figures 3A, B**.

**Table 2:** Antibacterial activity of plant extracts.

S. no.	Name of water borne bacterial pathogens	Antibacterial activity		
		IC_50_	MIC	MBC
1.	*Shigella dysenteriae*	0.237	0.625	1.25
2.	*Escherichia coli*	0.496	1.25	2.50
3.	*Salmonella typhimurium*	0.244	0.625	1.25
4.	*Vibrio cholera*	0.599	2.50	5
5.	*Klebsiella pneumonia*	0.351	1.25	2.50

In descending order, the effectiveness followed the sequence *S. dysenteriae* > *S. typhimurium* > *E. coli* > *K. pneumoniae* > *V. cholerae*. Based on the observations in **Figures 3A, B**, the extract showed the highest average percent growth inhibition at a concentration of 2.5 mg/mL. Furthermore, the IC_50_ values of the extract against the tested pathogens were found to be lowest for *S. dysenteriae*, indicating the strongest antibacterial activity (**Table 2**).

### Acute toxicity test

**Figure 4:**
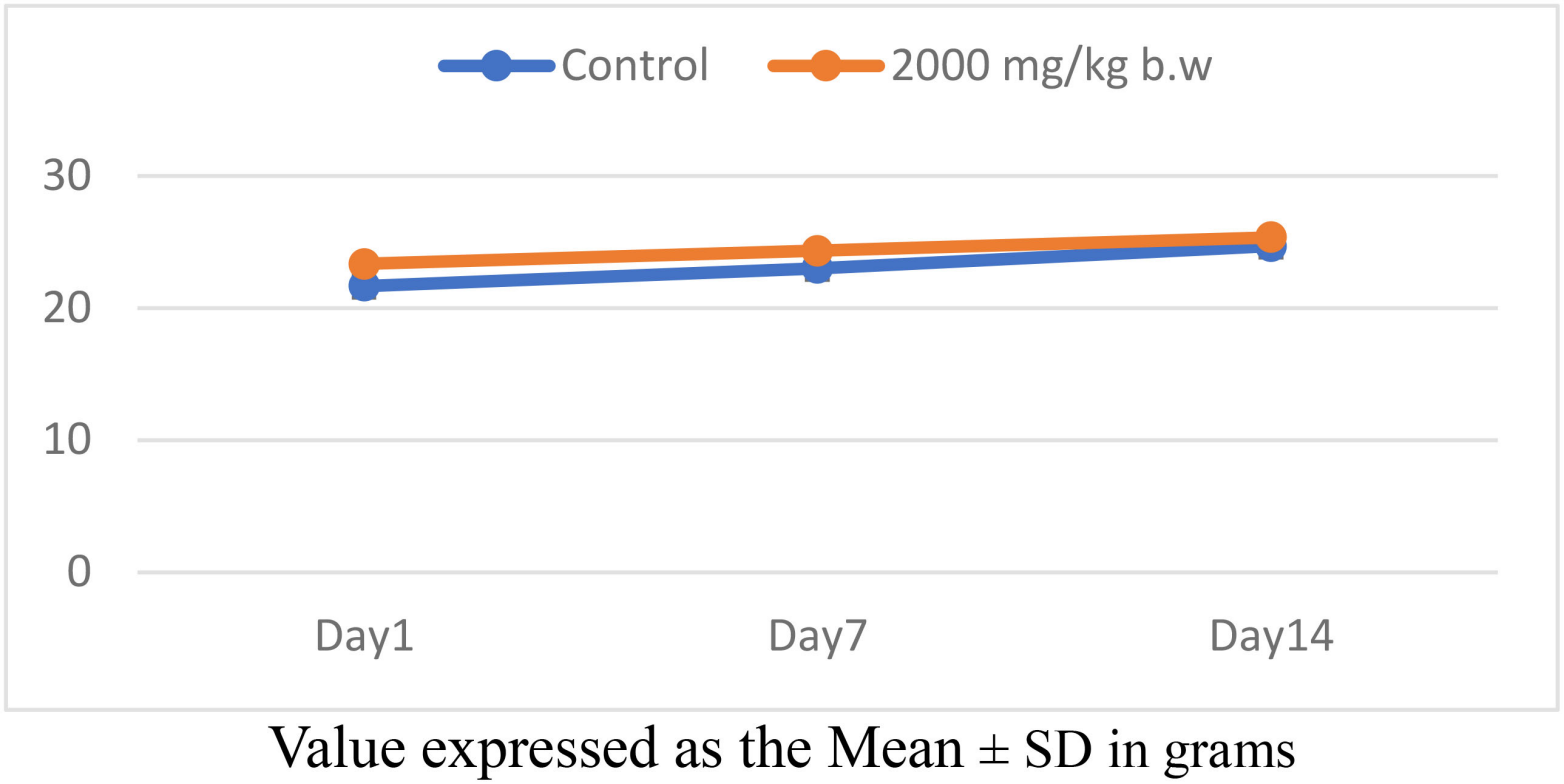
Mean body weight (g).

In the acute oral toxicity study, a single dose of 2,000 mg/kg b.w. of the methanolic leaf extract of *P. pinnata* was administered. The extract was found to be safe, as no mortality or observable signs of toxicity were recorded throughout the observation period. These findings indicate that the LD_50_ of the extract is greater than 2,000 mg/kg (**Table 3**). Furthermore, no significant differences were observed in body weight or relative organ weights between the control group and the treated group over 14 days (**Figure 4**).

**Table 3:** Relative organ weight (in terms of g/100 g).

Test Concentration	Lung	Liver	Kidney	Spleen
Control	0.677 ± 0.02	4.755 ± 0.26	1.363 ± 0.18	0.242 ± 0.06
2,000 mg/kg b.w.	0.701 ± 0.05	4.771 ± 0.17	1.328 ± 0.06	0.235 ± 0.02

Value expressed as the Mean ± SD in grams.

### Effect of plant extract on castor-oil-induced diarrhea

**Figure 5:**
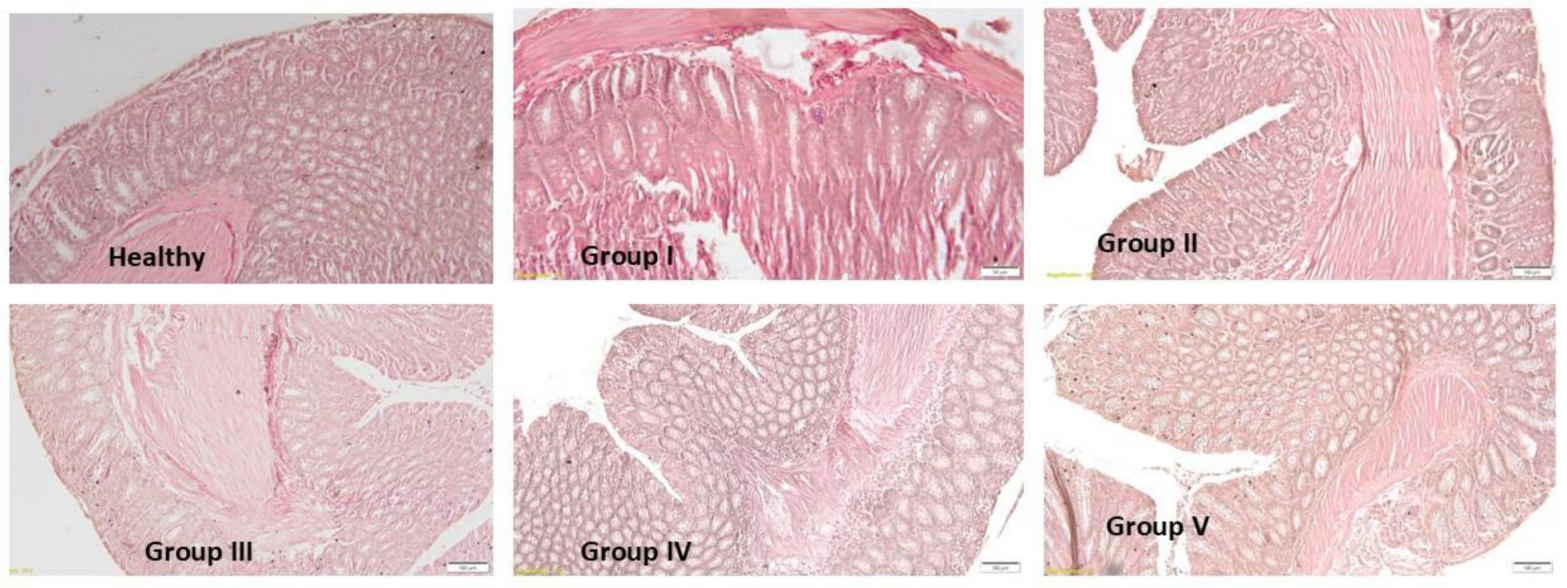
Histological appearance of colon under ×10 magnification. Group I, untreated castor oil; group II, loperamide + castor oil; group III, 200 mg/kg b.w. extract + castor oil; group IV, 400 mg/kg b.w. extract + castor oil; group V, 600 mg/kg b.w. extract + castor oil.

All tested doses of the *P. pinnata* extract significantly reduced the defecation frequency compared to the negative control. The mean number of fecal outputs at 200, 400, and 600 mg/kg doses was 15.00 ± 1.73, 12.67 ± 1.15, and 8.67 ± 1.53, respectively, while the loperamide-treated group showed 6.33 ± 2.08. The corresponding percent inhibition of total defecation was 22.4%, 34.45%, and 55.14% for the extract-treated groups and 67.25% for loperamide. Similarly, the mean number of diarrheal stools was reduced to 12.33 ± 2.31, 10.00 ± 1.00, and 7.67 ± 1.53 at the respective extract doses compared to 5.33 ± 1.53 in the loperamide group. Inhibition of diarrheal feces was 26.03%, 40.11%, and 53.98% for the extract, while loperamide achieved 68.02% inhibition (**Table 4**). To assess the significance of the chosen parameter, a correlation test was performed, as shown in **Figure 5**.

**Table 4:** Effect of *P. pinnata* leaf extract in mice.

Group	Treatment	Dose	Total no. of feces	% of inhibition of defecation	Total no. of diarrhea	% of inhibition of diarrhea
I	Castor oil	0.5 mL	19.33 ± 1.53	0	16.67 ± 0.58	0
II	Castor + LPM	3 mg/kg b.w.	6.33 ± 2.08	67.25	5.33 ± 1.53	68.02
III	Castor + extract	200 mg/kg b.w.	15.00 ± 1.73	22.4	12.33 ± 2.31	26.03
IV	Castor + extract	400 mg/kg b.w.	12.67 ± 1.15	34.45	10.00 ± 1.00	40.11
V	Castor + extract	600 mg/kg b.w.	8.67 ± 1.53	55.14	7.67 ± 1.53	53.98

Values are expressed as mean ± SD.

LPM, loperamide.

The statistical significance between castor-oil-induced diarrhea and castor-oil-induced diarrhea treated with plant extract at various concentrations was examined through ANOVA. The comparison involved the number of mouse feces, the percentage of defecation inhibition, the number of mice with diarrhea, and the overall percentage of diarrheal inhibition. The *P*-value was 0.02, indicating that a dose of 600 mg/kg b.w. significantly reduced the number of feces and the percentage of defecation inhibition in diarrheal mice, with a highly significant *P*-value (<0.05).

### Histopathology study

All of the colon histological sections (**Figure 5**) showed healthy, normal architecture and there were no significant pathological effects visible in either untreated group (e.g., no crypt distortion, epithelial sloughing, or infiltration indicative of diarrhea-inducing agents like castor oil). The effects were likely not histologically severe due to a short period of diarrhea induced on mice, i.e., 1-day treatment only.

### GC–MS analysis of *P. pinnata* plant extract

**Figure 6:**
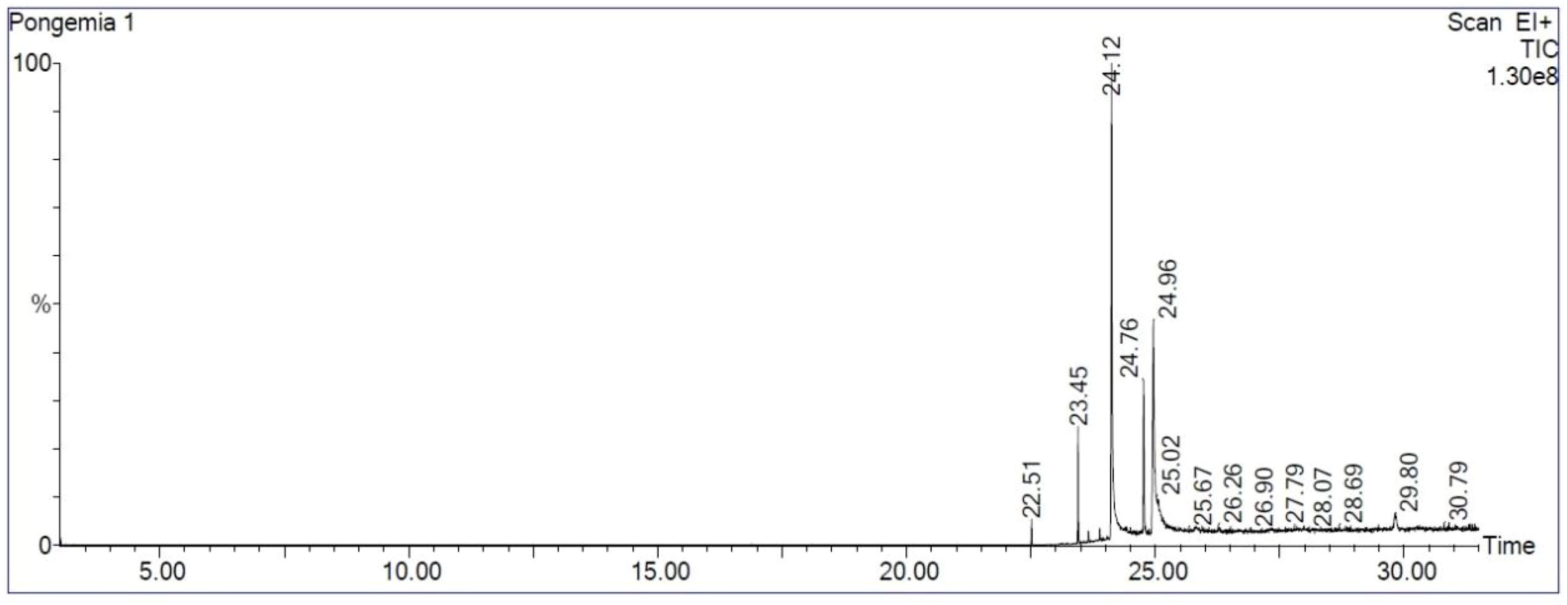
GC–MS chromatogram.

**Figure 7:**
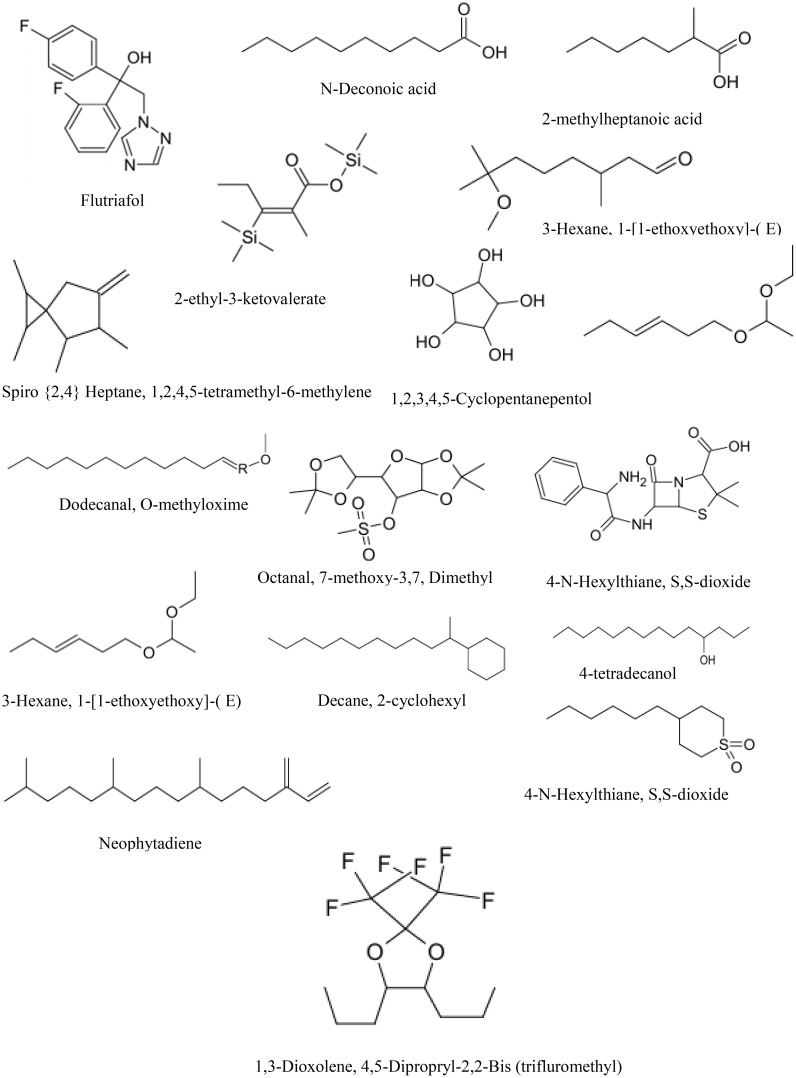
Molecular structure of bioactive molecules character*ized* from *P. pinnata*.

The GC–MS analysis of the plant showed the presence of 22 secondary metabolite compounds, as detailed in **Table 5**. The dominant bioactive compounds identified in the methanolic extract were N-decanoic acid (100%Norm), 4-N-hexylthiane (87.19%Norm), and neophytadiene (20.67%Norm), but trace amounts of bioactive compounds defined the overall potential of the plant. Norm% is a semi-quantitative measure, not an exact concentration. It reflects relative composition, not absolute amount (e.g., mg/mL). The characterization of these bioactive compounds depends on factors such as peak area, molecular weight, and molecular formula. The GC–MS chromatogram (**Figure 6**) and the molecular structures are illustrated in **Figure 7**.

**Table 5:** Identified secondary metabolites of *P. pinnata* leaf extract.

RT	Compound name	Area %	Mol. wt.	Formula
22.51	Spiro {2,4} heptane, 1,2,4,5-tetramethyl-6-methylene	0.535	164	C_12_H_20_
23.45	Neophytadiene	3.029	278	C_20_H_38_
23.65	Tran-(2-ethylcyclopentyl) methanol	0.456	128	C_7_H_14_O
23.88	2-Methylheptanoic acid	0.359	144	C_8_H_16_O_2_
24.12	N-decanoic acid	27.697	172	C_10_H_20_O_2_
24.302	1,2,3,4,5-Cyclopentanepentol	0.817	140	C_5_H_10_O_5_
24.382	1,2,5,6-Di-O-isopropylidene-3-O-methane sulfonyl glucofuranose	0.450	388	C_13_H_22_O_8_S
24.76	Neophytadiene	5.724	278	C_20_H_38_
24.96	4-N-Hexylthiane, S, S-dioxide	24.148	218	C_11_H_22_O_2_S
25.137	Decane, 2-cyclohexyl	2.004	172	C_16_H_32_
25.243	2-ethyl-3-ketovalerate; 2TMS derivative	0.908	288	C_7_H_12_O_3_
25.323	1,3-Dioxolane, 2-pentacyl-	0.500	284	C_18_H_36_O_2_
25.353	3-Hexane, 1-[1-ethoxyethoxy]-, (E)	0.346	288	C_10_H_20_O_2_
25.403	Isopropyl 5,11-dihydroxy-3,7,11-trimethyl-2-dodecenoate	0.396	314	C_18_H_34_O_4_
25.503	1,3-Dioxolane, 2-pentadecyl-	0.432	284	C_18_H_36_O_2_
25.813	4-Tetradecanol	0.664	214	C_14_H_30_O
26.263	7-Methoxy-3,7-dimethyloctanal	0.412	186	C_11_H_22_O_2_
27.328	1,3-Dioxolene, 4,5-dipropryl-2,2-bis (trifluromethyl)-	0.351	294	C_11_H_16_ F_6_O_2_
27.85	1,3-Dioxolene, 2-pentadecyl-	0.321	284	C_18_H_36_O_2_
28.30	Isopropyl 5,11-dihydroxy-3,7,11-trimethyl-2-dodecenoate	2.350	314	C_18_H_34_O_4_
29.82	Flutriafol	0.492	373	C_16_H_13_F_2_N_3_O
30.795	Dodecanal, O-methyloxime	0.371	213	C_13_H_27_NO

The compound identified included tran-(2-ethylcyclopentyl)methanol and dodecanal, with O-methyl oxime detected as a contaminant. The system also incorrectly identified some synthetic compounds, such as 1,3-dioxolane, 2-pentacyl-; 3-hexane, 1-[1-ethoxyethoxy]-, (E); and 1,3-dioxolane, 4,5-dipropyl-2,2-bis(trifluoromethyl)-, whose norm% is also very low. Additionally, 2-ethyl-3-ketovalerate, a chemically modified 2TMS (trimethylsilyl) derivative containing two trimethylsilyl (-Si(CH_3_)_3_) groups, was identified.

### *In silico* ADME prediction for drug likeness analysis of bioactive compounds

ADME property analysis is a powerful procedure for the assessment of potential drug molecules. Many factors, including pharmacokinetics, lipophilicity, water solubility, physicochemical characteristics, drug resemblance, and medicinal chemistry, are examined under the ADME study to determine which bioactive compounds are most likely to produce a powerful medication. The ADME results detailed in **Table 6** reveal that all bioactive compounds acquire acceptable drug-like properties in addition to the other drug likeness parameters. The physicochemical properties of all selected compounds satisfied Lipinski’s Rule of Five, like molecular weight (MW) <500 Da, number of H-bond donors (HBD) <5, number of H-bond acceptors (HBA) <10, and LogP value <5, etc., except decane, 2-cyclohexyl, and neophytadiene, which have LogP values more than 5. The majority of the molecules have high solubility in water, ranging from high solubility to moderate and low solubility depending on the structural properties.

**Table 6:** Lipinski properties of *P. pinnata* bioactive compounds calculated using SwissADME.

Molecule	MW	HBA	HBD	MR	TPSA	Log P	ESOL C	GIA	BBBP	log Kp (cm/s)	LV	Bio-rad
Flutriafol	301.29	5	1	76.03	50.94	2.69	Soluble	High	Yes	-6.5	0	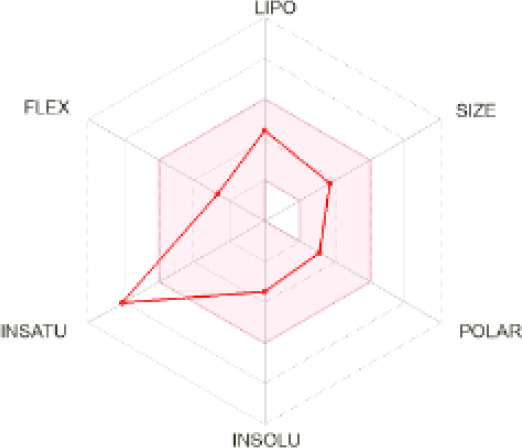
1,2,5,6-Di-O-isopropylidene-3-O-methansulfonyl glucofuranose	338.37	8	0	73.81	97.9	0.91	Very soluble	High	No	-8.3	0	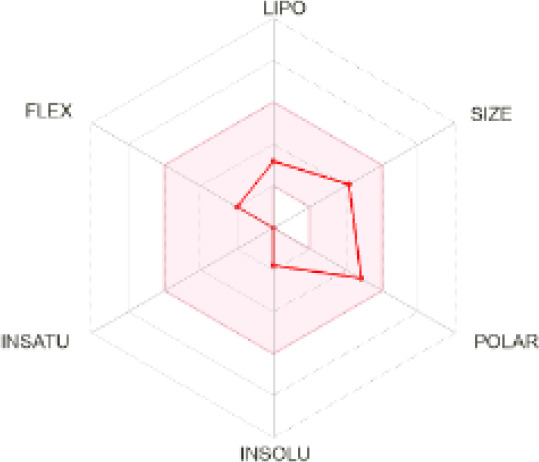
Spiro {2,4} Heptane, 1,2,4,5-tetramethyl-6-methylene	164.29	0	0	54.84	0	3.63	Soluble	Low	Yes	-4.75	0	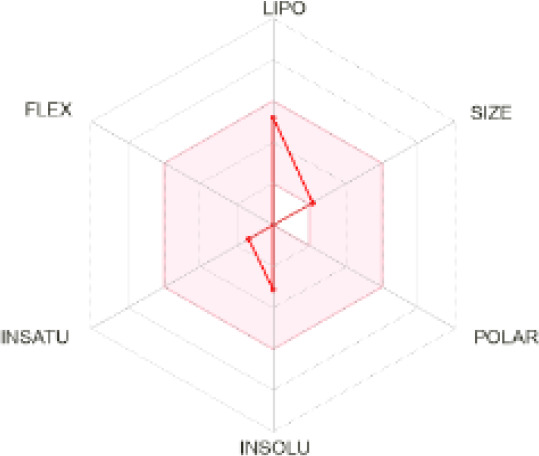
Ampicillin	349.4	5	3	92.56	138.03	0.08	Very soluble	Low	No	-1.33	0	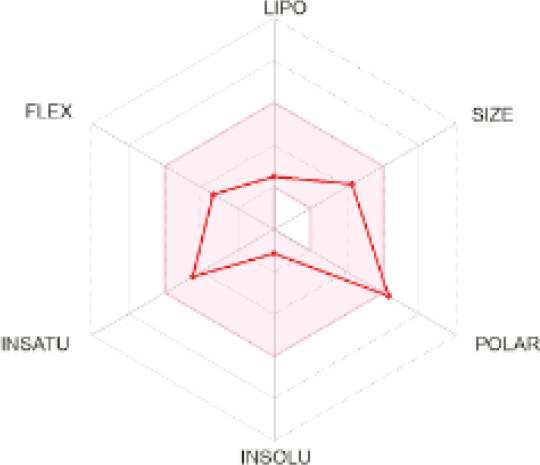
Decane, 2-cyclohexyl	252.48	0	0	86.53	0	6.68	Poorly soluble	Low	No	-9.23	1	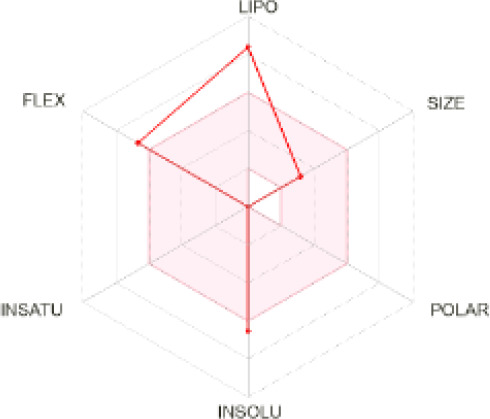
4-N-Hexylthiane, S,S-dioxide	218.36	2	0	61.84	42.52	3.02	Soluble	High	Yes	-5.08	0	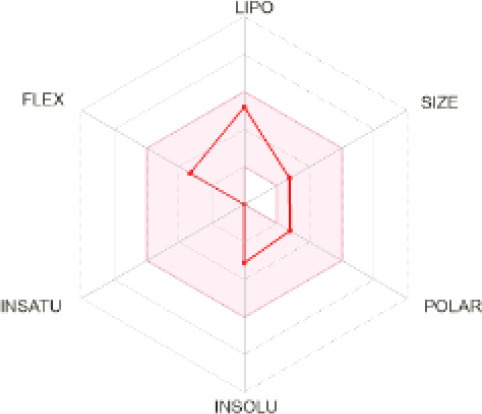
Isopropyl 5,11-Dihydroxy-3,7,11-Trimethyl-2-dodecenoate	314.46	4	2	91.81	66.76	3.68	Soluble	High	Yes	-5.39	0	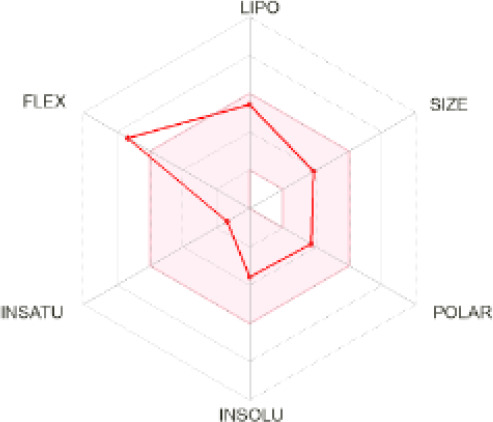
1,2,3,4,5-Cyclopentanepentol	150.13	5	5	29.84	101.15	-2.21	Highly soluble	Low	No	-9.41	0	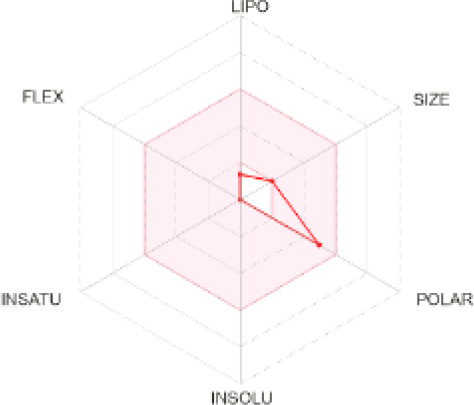
N-Deconoic acid	172.26	2	1	51.96	37.3	3	Soluble	High	Yes	-4.45	0	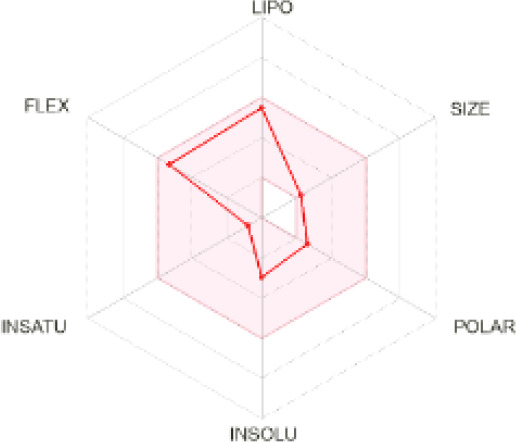
4-tetradecanol	214.39	1	1	70.57	20.23	4.62	Moderately soluble	High	Yes	-3.38	0	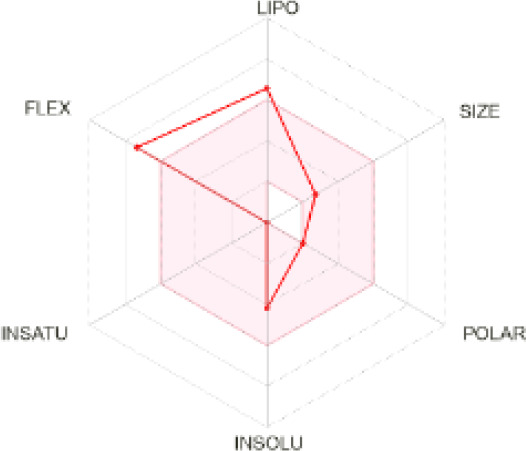
2-methylheptanoic acid	144.21	2	1	42.34	37.3	2.14	Soluble	High	Yes	-5.13	0	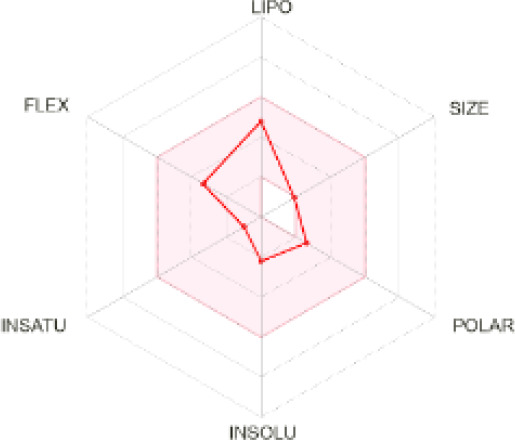
2-ethyl-3-ketovalerate;	288.53	3	0	82.24	35.53	3.19	Moderately soluble	High	Yes	-4.66	0	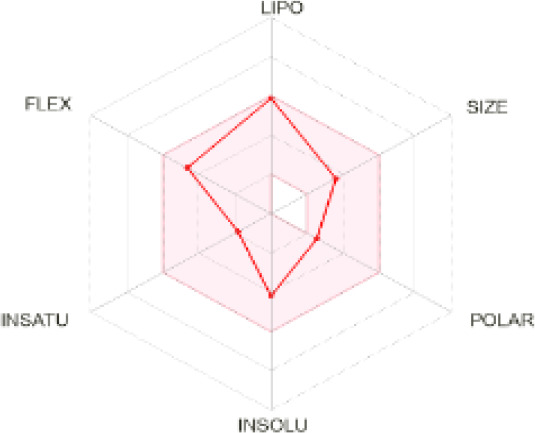
Tran-(2-ethylcyclopentyl) methanol	128.21	1	1	39.62	20.23	2.01	Very soluble	High	Yes	-5.41	0	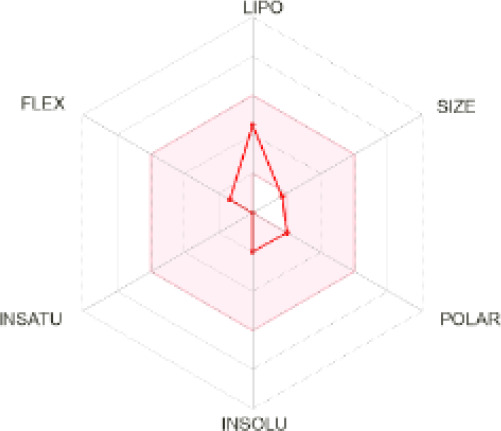
3-Hexane, 1-[1-ethoxyethoxy]-( E)	172.26	2	0	51.88	18.46	2.6	Soluble	High	Yes	-5.5	0	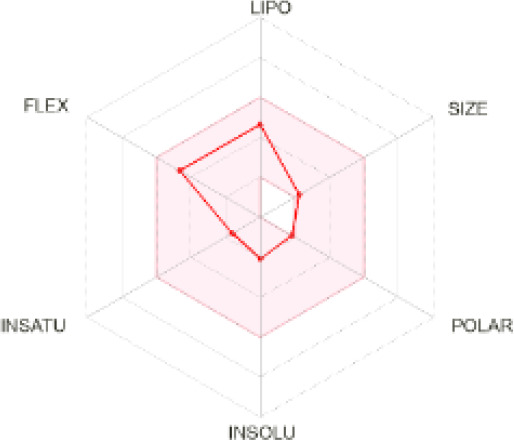
Neophytadiene	278.52	0	0	97.31	0	7.07	Poorly soluble	Low	No	-1.17	1	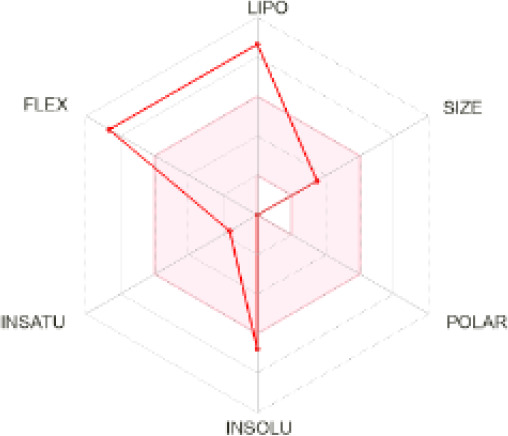
Dodecanal, O-methyloxime	213.36	2	0	69.37	21.59	4.41	Soluble	High	Yes	-3.72	0	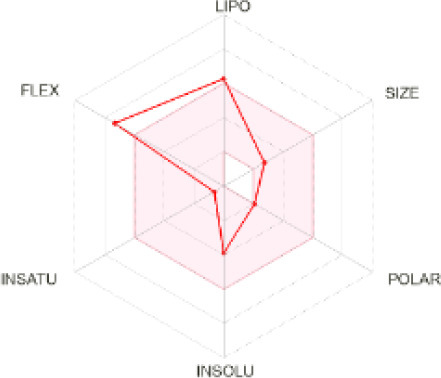
Isopropyl 5,11-Dihydroxy-3,7,11-Trimethyl-2-dodecenoate	314.46	4	2	91.81	66.76	3.68	Soluble	High	Yes	-5.39	0	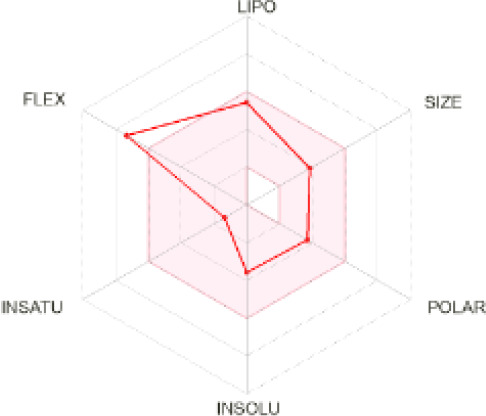
Octanal, 7-methoxy-3,7, Dimethyl	186.29	2	0	56.31	26.3	2.51	Very soluble	High	Yes	-5.89	0	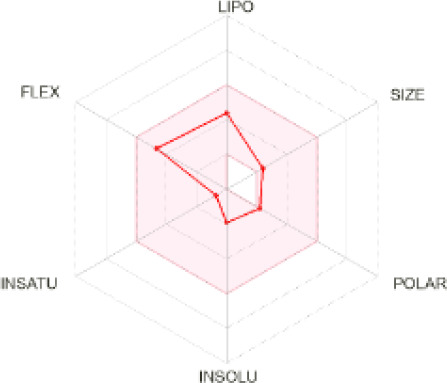
1,3-Dioxolene, 4,5-Dipropryl-2,2-Bis (trifluromethyl)-	294.23	8	0	55.47	18.46	4.35	Moderately soluble	Low	No	-4.86	0	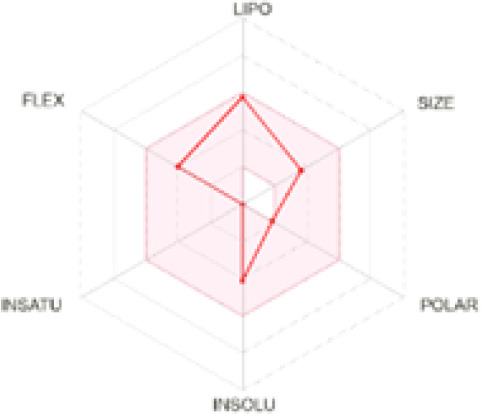

Every molecule has a topological polar surface area (TPSA) of less than 140, and notably, the logKp value, which signifies skin permeability, falls within the usual range of -8.0 to -1.0, indicating that the molecules might have potential to inhibit the enzymes (**Table 6**). To facilitate a rapid assessment of drug likeness, the bioavailability radar (Bio-rad) is also displayed (where the size, polarity, solubility, flexibility, saturation, and lipophilicity are examples of physicochemical properties that are taken into consideration. A molecule’s radar plot must totally fall within this area in order to be categorized as drug like, and this is indicated by a pink area representing the descriptors used to establish the physicochemical range on each axis. While most of the molecules may be subject to additional investigation as potential drugs based on ADME characteristics and the computation of molecular characteristics and bioactivity using Lipinski’s Rule of Five, here we highlight the three molecules—flutriafol, 1,2,5,6-di-O-isopropylidene-3-O-methansulfonyl glucofuranose, and spiro{2,4}heptane, 1,2,4,5-tetramethyl-6-methylene, which have a higher binding affinity score than the widely used drug ampicillin.

### Bacterial protein receptor identification against ampicillin

**Figure 8:**
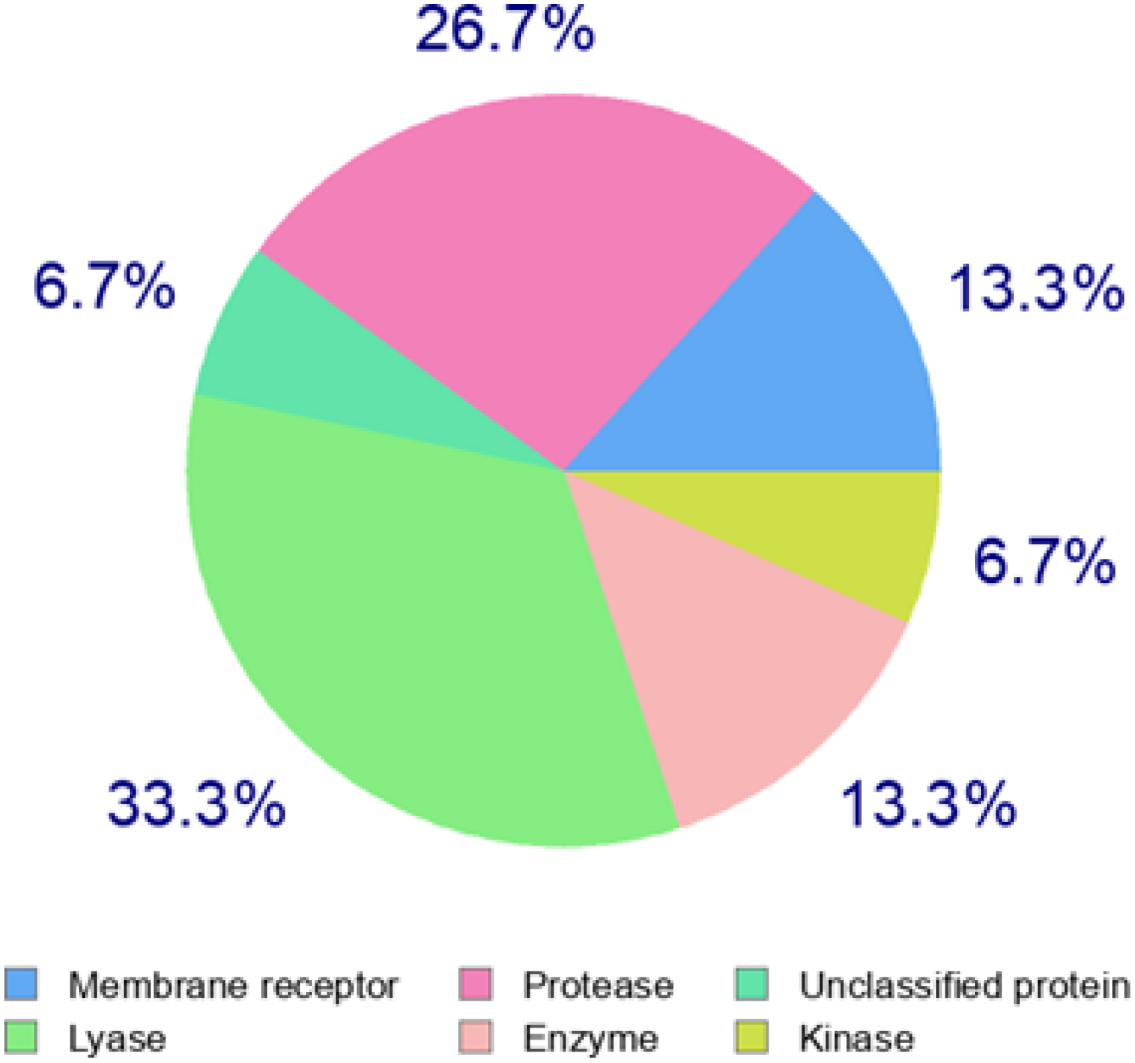
Identification of receptor protein against the metabolites of *P. pinnata*.

After looking through relevant targets in the Swiss Target Prediction database, 535 potential *P. pinnata* bioactive compound targets were identified. Among them, 74 (13.3%) targets belong to membrane receptors (**Figure 8**). Therefore, here we select the penicillin-binding protein (PBP), one of the most significant membrane receptors that play a key role in the biosynthesis of plasma membrane and cell wall. The reason for selecting the PBP receptor was that we used ampicillin as an antibiotic to control the growth of bacteria in our *in vivo* study.

### Molecular docking and interaction analysis

**Figure 9:**
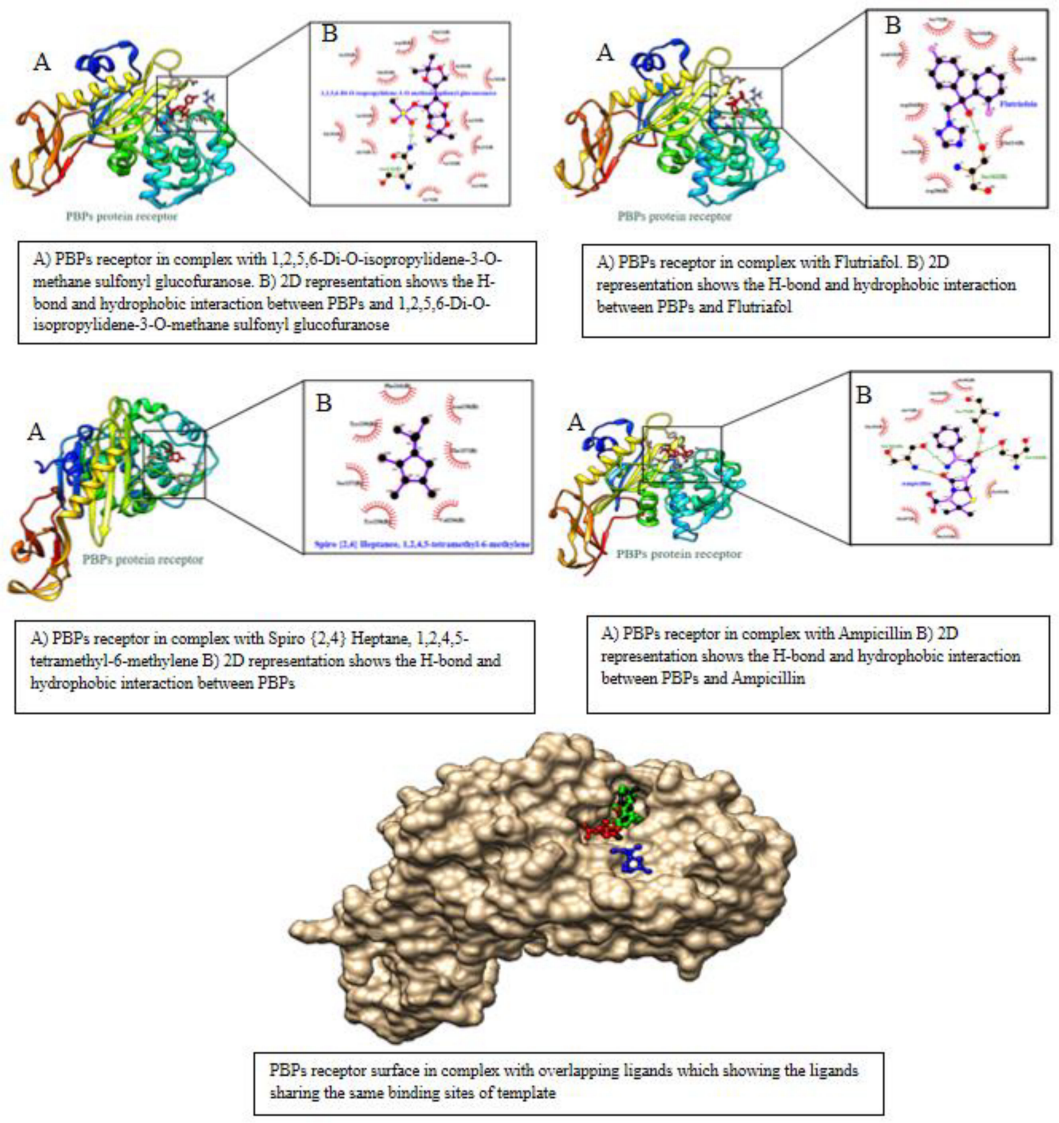
Molecular docking complex diagram.

Docking of the ligand molecules (bioactive compounds) was done at a particular location on the PBP’s surface. The Autodock vina tools were used to dock the described bioactive compounds, and the results were analyzed in terms of binding affinity (Gibbs free energy, ΔG). The docking analysis results for the bioactive compounds and known PBP inhibitors are shown in **Table 7**. Here it was observed that flutriafol, 1,2,5,6-di-O-isopropylidene-3-O-methansulfonyl glucofuranose, and spiro{2,4}heptane, 1,2,4,5-tetramethyl-6-methylene showed higher binding affinities of -8.1, -6.7, and -6.6, respectively, which were comparatively stronger than the standard drug ampicillin, which has a binding affinity of only -6.3. The docking pose of the molecules, which showed a lower ΔG score than ampicillin, is presented in **Figure 9**. An analysis of the docked structures of bioactive compounds using the structure visualization tool Pymol confirmed that all ligand molecules were docked in the same active pocket of the PBP receptor. Furthermore, molecular docking was analyzed in terms of molecular interactions, such as hydrogen bonds, hydrophobic interactions, and amino acid residues contributing to the interactions presented in **Table 8**.

**Table 7:** Results of molecular docking.

Protein receptor	Ligands	Binding energy (ΔG), kcal/mol
PBP’s receptor	Flutriafol	-8.1
	1,2,5,6-Di-O-isopropylidene-3-O-methansulfonyl glucofuranose	-6.7
	Spiro {2,4} heptane, 1,2,4,5-tetramethyl-6-methylene	-6.6
	Decane, 2-cyclohexyl	-6.4
	Ampicillin	-6.3
	4-N-Hexylthiane, S,S-dioxide	-6
	2-(2-Cyclohexyl-2-triethylsilyloxy-1-hydroxyethyl)-3-methyl-1,4-dioxaspiro[5.4]decane	-5.5
	Isopropyl 5,11-dihydroxy-3,7,11-trimethyl-2-dodecenoate	-5.3
	1,2,3,4,5-Cyclopentanepentol	-5.2
	N-Deconoic acid	-5.1
	4-Tetradecanol	-4.9
	2-Methylheptanoic acid	-4.7
	2-Ethyl-3-ketovalerate;	-4.7
	Tran-(2-ethylcyclopentyl) methanol	-4.6
	3-Hexane, 1-[1-ethoxyethoxy]-(E)	-4.4
	Neophytadiene	-3.9
	Dodecanal, O-methyloxime	-3.8
	1,3-Dioxolane, 2-pentacyl-	-3.4
	Isopropyl 5,11-dihydroxy-3,7,11-trimethyl-2-dodecenoate	-3.02
	Octanal, 7-methoxy-3,7, dimethyl	-2.95
	1,3-Dioxolene, 4,5-dipropryl-2,2-bis (trifluromethyl)-	-2.93

**Table 8:** Molecular interaction analysis between bioactive compounds and PBPs.

Protein receptor	Ligand name	Hydrogen bond	Hydrophobic interaction
PBPs	Flutriafol	Glu346, Arg350, Thr563, Lys583,	Glu349, Ala345, Thr342, Phe452
	1,2,5,6-Di-O-isopropylidene-3-O-methansulfonyl glucofuranose	Glu599, Glu357	Arg350, Asp353, Tyr598, Val596
	Spiro {2,4} heptane, 1,2,4,5-tetramethyl-6-methylene	–	Tyr498, Tyr515,Thr629, Thr654, Glu686 Glu687, Tyr690
	Ampicillin	Asp453, Thr563, Gln566, Lys583	Glu349, Phe452, Lys455, Glu545, Glu561

Some extremely beneficial hydrophobic interactions were part of the binding mode of ligands in the active site of the PBP receptor, which was analyzed and shown in a 2D representation (**Figure 9**). These hydrophobic interactions were calculated within a 4-A° region surrounding the ligand molecules. The involvement of amino acids like Arg350, Asp353, Glu346, Lys583, Thr563, and Tyr690 was significant in the formation of H-bonds or hydrophobic contacts; similarly, the same set of amino acid residues was implicated in ampicillin binding to the PBP protein, too.

### Protein–protein interaction and functional analysis of receptor protein

**Figure 10:**
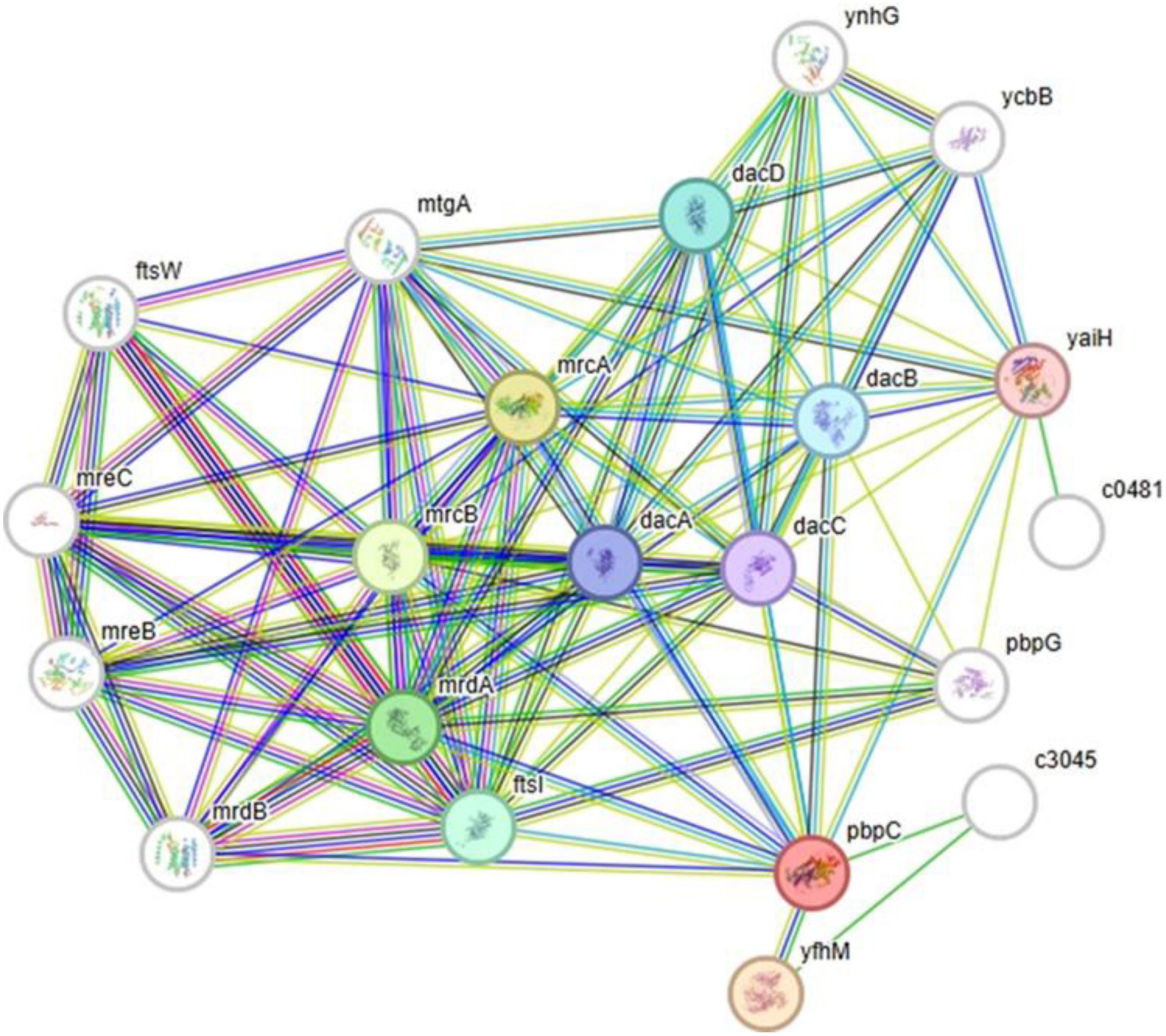
Protein–protein interaction analysis.

A protein–protein interaction analysis was carried out using the STRING database, which provides information regarding the physical and functional interactions of individual proteins. Understanding of single proteins is well established in terms of their function, but their interactions with other proteins are still undistinguishable. Here STRING analysis showed that PBP receptors interacted with several known or anticipated functional cohort proteins. Therefore, to identify the closest association between PBPs and other proteins, the network consisted of 21 nodes and 106 edges. The network was visualized using Cytoscape and further examined to determine the central genes and the genes associated with the central gene. The central genes were predicted using CytoHub, a plug-in tool that showed the significant role of central genes in the network and helped to choose the primary targets. The top 10 core target genes were yaih, dacB, dacC, dacA, mrcB, mrdA, ftsl, mrdB, c3045, and yfhM. A protein–protein interaction analysis (**Figure 10**) shows the direct interactions of pbpC (also known as PBP3 in some species like *E. coli*), such as pbpC-yaih, pbpC-dacB, pbpC-dacC, pbpC-dacA, pbpC-mrcB, pbpC-mrdA, pbpC-ftsl, pbpC-mrdB, pbpC-c3045, and pbpC-yfhM. Whereas several indirect interactions of pbpC were observed, these interactions were associated with several other proteins that modulate various biological processes. Function enrichment analysis indicates that peptidoglycan biosynthetic process, regulation of cell shape, and cell wall organization are the most important among the BPs. These BPs are mainly involved in cell membrane and cell wall formation in bacteria.

### Analysis of GO function and KEGG pathway enrichment

A total of 1,396 GO items were identified, including 614 BP (biological processes) and 243 MF (molecular functions). The significant BP and MF are shown in **Tables 9**, **10**, respectively. The BP results showed that the activity of active bioactive compounds of *P. pinnata* in diarrheal conditions was mostly focused on the regulation of plasma membrane and cell wall biosynthesis. The MF mainly included the mechanisms involved in carboxypeptidase activity, peptidoglycan glycosyltransferase activity, and penicillin binding (**Table 10**). To a certain extent, the various GO functions may also help to explain why the compounds of *P. pinnata* are effective in treating diseases like diarrhea. The KEGG pathway enrichment analysis revealed that the compounds were primarily involved in 13 signaling pathways (*p* < 0.01), and peptidoglycan biosynthesis and beta-lactam resistance are the top two significantly enriched pathways, which are shown in **Table 11**. Proteoglycan biosynthesis and membrane biosynthesis are the primary signaling pathways found to be enriched, including various genes such as ftsI, mrcB, dacA, mrdA, dacC, dacD, pbpC, dacB, mtgA, and mrcA.

**Table 9:** GO-term-associated PBPs.

GO	Gene ontology terms	Gene count	Background gene count	Strength	Signal	False discovery rate
GO:0009252	Peptidoglycan biosynthetic process	14	40	1.95	6.17	3.30E-21
GO:0008360	Regulation of cell shape	12	37	1.92	5.42	2.89E-18
GO:0071555	Cell wall organization	10	56	1.66	3.43	1.08E-12
GO:0006508	Proteolysis	10	98	1.42	2.42	1.47E-10
GO:0050789	Regulation of biological process	13	461	0.86	1	1.06E-07
GO:0043170	Macromolecule metabolic process	15	756	0.71	0.76	2.63E-07
GO:1901564	Organonitrogen compound metabolic process	15	755	0.71	0.76	2.63E-07

**Table 10:** Molecular function associated with PBPs.

GO ID	Term description	Gene count	Background Gene Count	Strength	Signal	False discovery rate
GO:0004180	Carboxypeptidase activity	12	19	2.21	6.94	4.92E-20
GO:0004185	Serine-type carboxypeptidase activity	11	15	2.27	6.8	7.29E-19
GO:0009002	Serine-type D-Ala-D-Ala carboxypeptidase activity	8	8	2.41	5.51	3.19E-14
GO:0008955	Peptidoglycan glycosyltransferase activity	7	7	2.41	4.8	2.55E-12
GO:0008658	Penicillin binding	5	5	2.41	3.32	1.71E-08
GO:0016740	Transferase activity	9	627	0.57	0.37	0.0253
GO:0071972	Peptidoglycan L,D-transpeptidase activity	2	6	1.93	0.67	0.0293
GO:0003824	Catalytic activity	15	1865	0.31	0.25	0.0474

**Table 11:** KEGG pathways associated with PBPs.

Term ID	Term description	Gene count	Background gene count	Strength	Signal	False discovery rate
ecc00550	Peptidoglycan biosynthesis	10	23	2.05	5.3	6.41E-16
ecc01501	beta-Lactam resistance	3	22	1.54	0.87	0.0065

**Figure 11:**
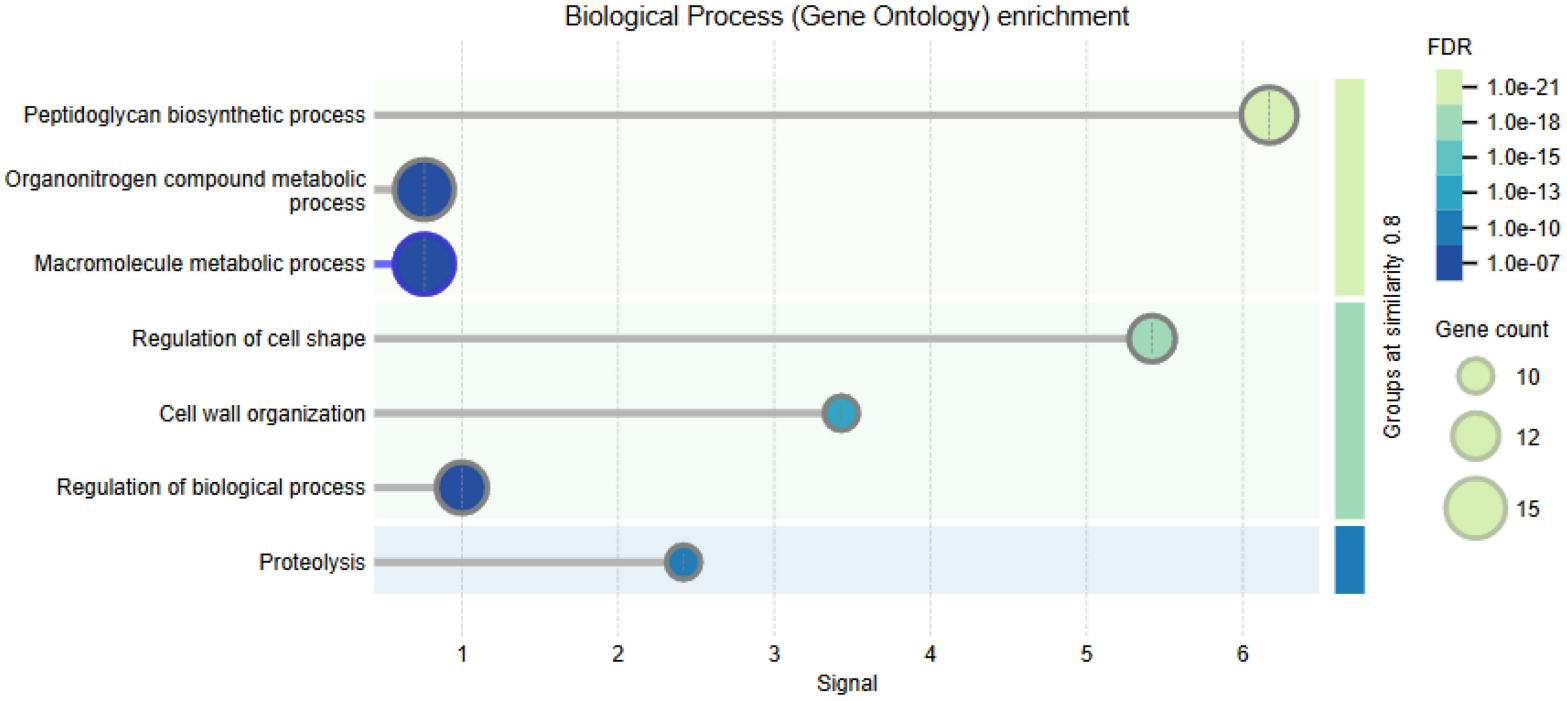
Significantly enriched biological process in diarrheal condition.

In the table, the gene count indicates how many proteins in your network are annotated with a particular term, and the background gene count indicates how many proteins in total (in your network and in the background) have this term assigned. The strength is the ratio of the number of proteins in the network annotated with a term to the number expected to be annotated with the same term in a random network. The signal is defined as the weighted harmonic mean of the observed/expected ratio and the -log of the false discovery rate (FDR) (**Figure 11**). FDR tends to emphasize larger terms because they can achieve lower *p*-values, while the observed/expected ratio highlights smaller terms, which have a high foreground-to-background ratio but cannot achieve low FDR values due to their size (indicating how significant the enrichment is) (*p*-values were corrected for multiple testing using the Benjamini–Hochberg method).

### Evaluation of the binding stability of protein–ligand complexes

**Figure 12:**
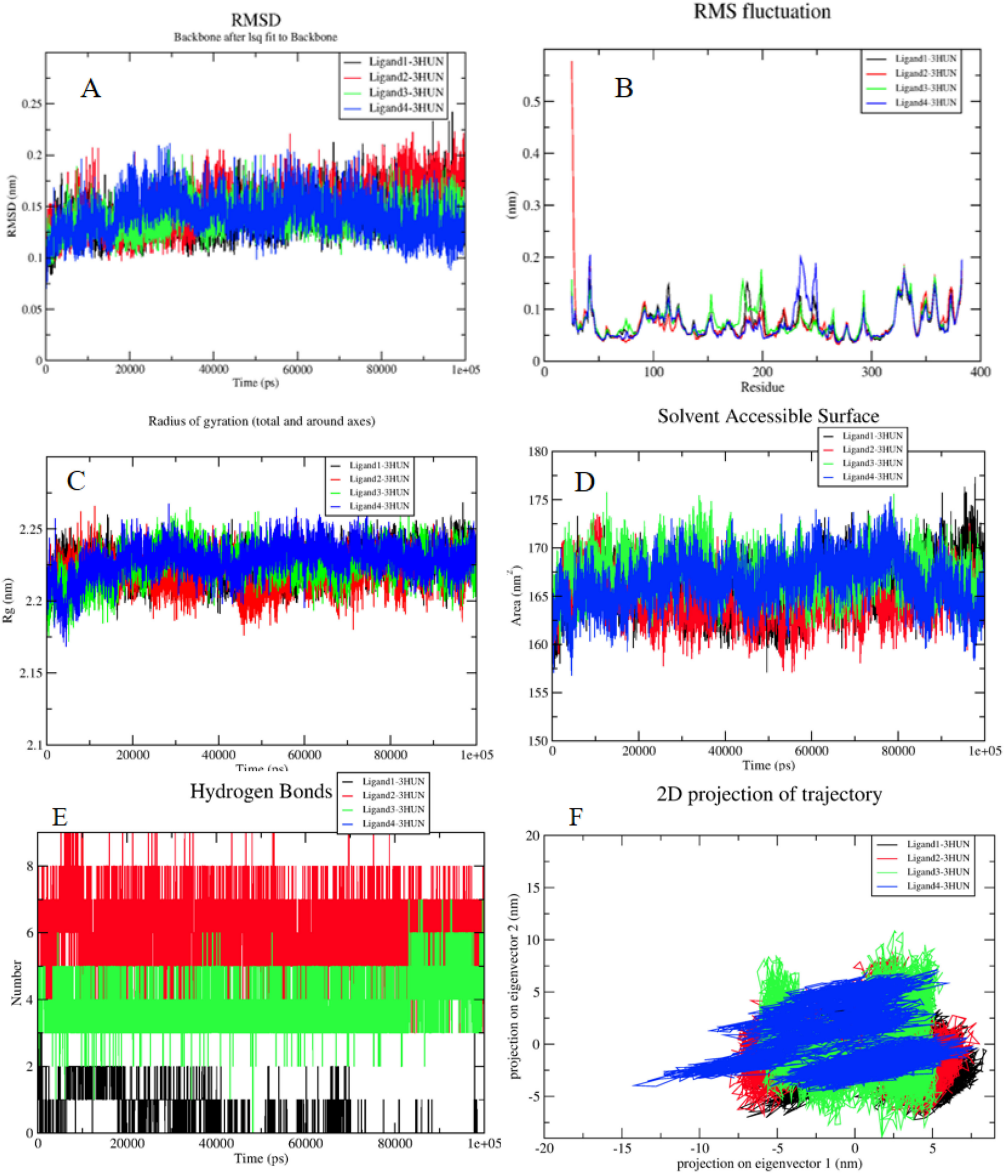
Molecular dynamics simulation trajectory analysis of protein–ligand complexes. **(A)** RMSD. **(B)** RMSF. **(C)** Radius of gyration (Rg). **(D)** SASA. **(E)** H-bond. **(F)** PCA of simulated trajectories of PBPs in complex with ligands. The black color represents the structural deviation of PBPs in complex with 1,2,5,6-dio-isoproglyidene-3-O-methane (Ligand1). The red color shows the structural deviation of PBPs in complex with ampicillin (Ligand2). The green color shows the structural deviation of PBPs in complex with flutriafol (Ligand3). The blue color shows the structural deviation of PBPs in complex with spiro{2,4}heptane, 1,2,4,5-tetramethyl-6-methylene (Ligand4).

A comparative analysis of all simulated system trajectories of protein in complex with control ligand ampicillin and flutriafol, 1,2,5,6-Di-O-isopropylidene-3-O-methane sulfonyl glucofuranose, and spiro{2,4}heptane, 1,2,4,5-tetramethyl-6-methylene was performed in terms of root mean square deviation (RMSD), root mean square fluctuation (RMSF), radius of gyration (Rg), solvent accessible surface area (SASA), hydrogen bond (H-bond), and principal component analysis (PCA) throughout the simulation time. MD simulation studies were used to compare the binding of selected ligands, including a known inhibitor, membrane-bound penicillin-binding proteins (PBPs). The RMSD analysis of simulated protein backbone trajectories for all protein–ligand complexes showed a slight increase at the initial stages, which was subsequently stabil*ized* during the MD simulation (**Figures 12A–F****).**

The comparative RMSD analysis of all simulated trajectories suggested that ligand binding stabilizes the PBPs’ structure. The average RMSD of the backbone was observed at nearly 2 Å for all of the complexes with different ligands. The RMSF analysis was performed to compare the flexibility behavior of protein residues after ligand binding. The RMSF of the C-alpha atoms of the protein was higher when ampicillin bound to PBPs at the initial residues; thereafter, fluctuations were lower during the simulation. The PBP–flutriafol (green color) complex showed less residual fluctuation throughout the simulation, except for residue numbers approximately 148–160, 185–190, and 195–205, which fluctuated from 0.75 to 2 Å. However, a similar pattern of residual fluctuations was observed in the PBP receptor upon binding spiro{2,4}heptane, 1,2,4,5-tetramethyl-6-methylene, with slight fluctuations near residues 130–150, extending up to 2 Å. From this analysis, it could be drawn that the binding of ligand molecules normal*ized* the PBPs’ structural movement during the course of simulation (**Figure 12B**).

Protein structure compactness and stability can correctly be defined in terms of the radius of gyration (Rg). The average Rg value for all of the protein–ligand complexes was depicted as nearly 2.2–2.25 nm by smaller changes from the initial to the end of the MD simulation. A comparative study of Rg suggested that all protein–ligand complexes exhibited a similar level of structural stability to that observed with the standard drug ampicillin with PBPs (**Figure 12C**). On the other hand, the decline in the total SASA score confirmed the reduced accessibility of amino acid residues to solvent and further compaction upon ligand binding. It was quietly evident in the case of PBP–flutriafol (Ligand3) and PBP–spiro{2,4}heptane, 1,2,4,5-tetramethyl-6-methylene (Ligand4) (**Figure 12D**). Hence, a decrease in the total SASA score indicated a decrease in the number of intramolecular hydrogen bonds from the surrounding environment. The number of hydrogen bonds during MD simulations indicates the stability of ligand binding at the surface throughout the simulation. For all of the protein–ligand complexes, the simulated trajectories were analyzed, and the number of H-bonds was observed (**Figure 12E**).

Throughout the MD simulation, the H-bond interaction between the ligands and residues of PBPs was observed, where the ampicillin–PBPs complex at 5,000 ps (5 ns) reached up to nine H-bonds, and due to continuous fluctuations throughout the entire simulation period, the H-bonds retained a count of eight at the end of the trajectory. Whereas the flutriafol–PBP complex system initially had five H-bonds, H-bonding fluctuated over the course of the MD simulation and, by the end, stabil*ized* at seven H-bonds. However, the analysis of PBP–1,2,5,6-di-O-isopropylidene-3-O-methane sulfonyl glucofuranose (Ligand1) and PBP–spiro{2,4}heptane, 1,2,4,5-tetramethyl-6-methylene (Ligand4) trajectories showed less H-bond connection between protein–ligand complexes, and at the end of the MD simulation, no H-bonds were observed. In fact, the H-bond analysis revealed that flutriafol has a more or less equal impact on conformational changes and stability compared to ampicillin, which might play a major role in defining a better binding affinity toward PBPs. The structural snapshots from the MD trajectory were extracted every 2 ps, yielding a set of eigenvectors that provided a vectorial representation of each component of the motion, thus revealing its direction.

The eigenvectors of the covariance matrix of the simulated system were calculated, and trajectories were filtered according to each eigenvector, which can be analyzed and represented in the plot, showing the patterns of dominant atomic motions of macromolecules (**Figure 12E**). A few low-frequency eigenvectors with large eigenvalues often explain the overall oscillations of macromolecules. The eigenvectors and eigenvalues resulting from distinct trajectories should be similar to one another if the movements are similar. Since many of the internal motions are captured by the first few eigenvectors, the first two (EV1 and EV2) in each case accounted for a sizable percentage of the overall motion. The projection of trajectories onto the first two principal components depicted the receptor’s motion in protein–ligand complexes in phase space (as shown in **Figure 12F**). According to the pictorial representation, the PBP–spiro{2,4}heptane, 1,2,4,5-tetramethyl-6-methylene (Ligand4) complex showed greater atom scattering than the PBP–ampicillin complex, indicating more conformational changes in the crystal structure, which was consistent with the MD analysis. Similarly, the PBP receptor showed lower atomic movement upon binding to flutriafol (Ligand3) and 1,2,5,6-di-O-isopropylidene-3-O-methane sulfonyl glucofuranose (Ligand1), indicating greater binding stability with all of the selected ligands.

## Discussion

Medicinal plants offer a valuable reservoir of potential chemotherapeutic agents. *P. pinnata* plants often contain alkaloids, phenols, and terpenoids that can have therapeutic implications. The success of natural products depends not only on the presence of diverse bioactive compounds but also on their concentrations. Some metabolites, such as flavonoids and terpenoids, tend to associate well with phosphatidylcholine due to their hydrophobic and phenolic properties (Kumar, 2011). However, the effectiveness of plant extracts and phyto-molecules is constrained by poor lipid solubility and improper molecular size, leading to limited bioavailability and *in vivo* effects.

Numerous medicinal and aromatic plants are recogn*ized* for their antioxidant properties, which are crucial for safeguarding our bodies against various ailments. In line with the traditional healer’s ethnomedicinal claims, our study delved into the radical scavenging properties and reducing power assay of the methanolic extract from *P. pinnata*. Notably, this extract demonstrated substantial inhibition of DPPH and superoxide radical scavenging activities, alongside robust reducing power. A diminished IC_50_ (half-maximal inhibitory concentration) indicates a heightened capacity to neutralize free radicals, while an elevated IC_50_ signifies diminished scavenging effectiveness, as a greater quantity of scavengers is necessary to attain 50% scavenging efficacy.

The ethanolic extract of *P. pinnata* bark displayed superior radical scavenging activity compared to the leaves, with 54.8% inhibition at a bark concentration of 0.5 mg/mL (Sagar and Kumud, 2013). A comparative analysis of methanol extracts from *P. pinnata* leaves and seeds was likewise conducted at concentrations of 100–600 μg/mL using the DPPH assay, which resulted in IC_50_ values of 116.88 and 112.36 μg/mL, respectively (Pande et al., 2014). Additionally, *P. pinnata*’s high flavonoid content contributes to potent antioxidant and radical scavenging activities, which are especially evident in its seed oil (Shafi et al., 2020; Nataraj, 2021).

Moving forward, the methanolic extract of *P. pinnata* leaves exhibited robust antibacterial activity. Notably, it exerted potent inhibitory effects against *Staphylococcus. aureus*, *Proteus vulgaris*, *Staphylococcus epidermidis*, *E. coli*, *Pseudomonas aeruginosa*, *Enterobacter aerogenes*, and *Salmonella enterica sarovar* Typhimurium, with mild inhibitory effects against *S. enterica sarovar* Typhi and *K. pneumoniae* (Arote et al., 2009; Sharma et al., 2021). Similarly, the seed oil of *P. pinnata* demonstrated toxicity against *Yersinia enterococcai*, *Listeria monocytogenes*, *E. coli*, and *Salmonella paratyphi* (Kesari et al., 2009; Rajput et al., 2021), which aligned with our findings.

**Figure 13:**
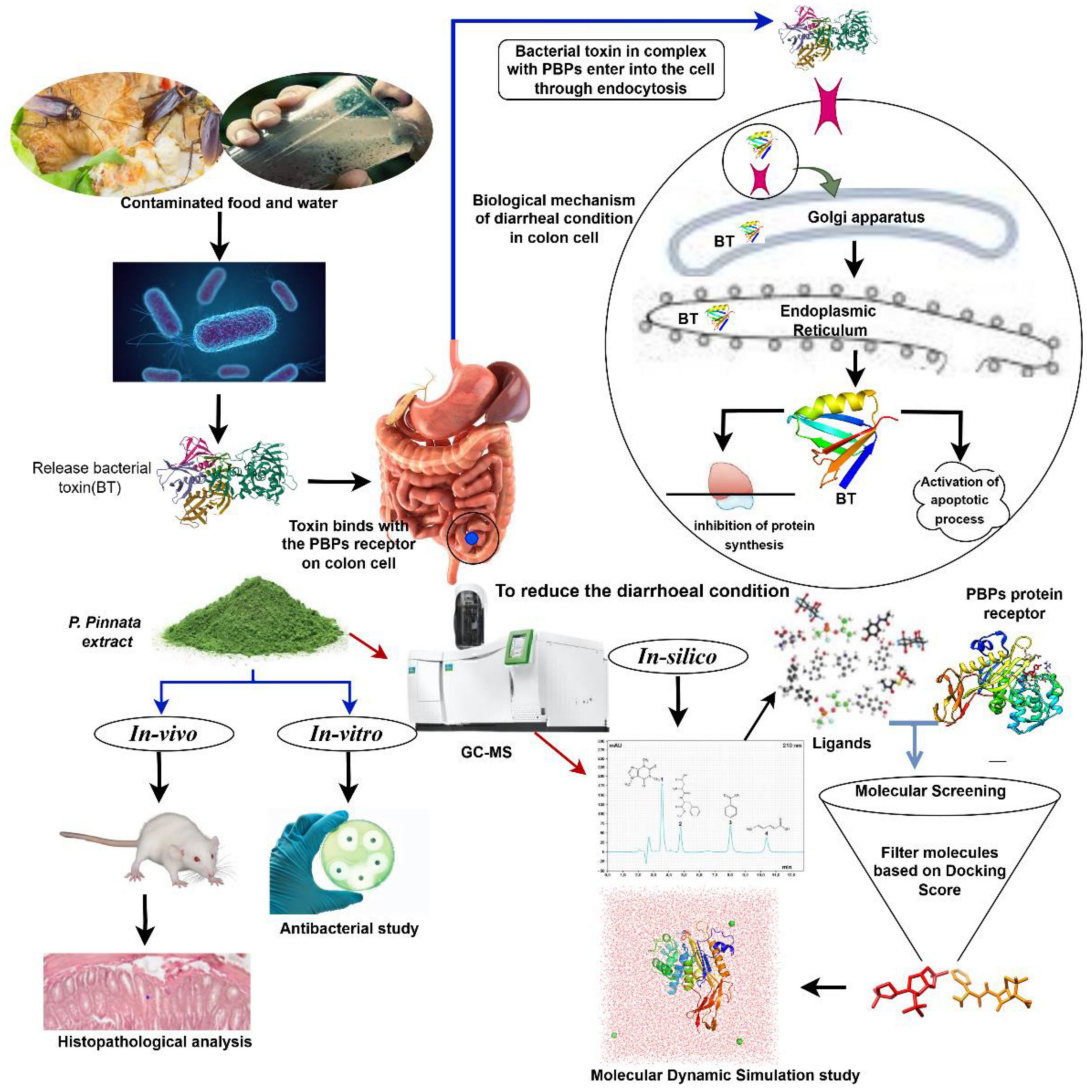
Validation of *in vitro* antibacterial activity of *P. pinnata* with their bioactive compounds using *in silico* approach to bind the responsible bacterial toxin (causing diarrhea).

The variation in MICs highlights differences in bacterial cell wall composition, particularly in gram-negative bacteria with their outer membrane, a significant barrier for bioactive molecules (Olusola-Makinde and Bayode, 2021). The interaction of bioactive compounds with bacterial cell membranes can disrupt their structures, potentially increase the permeability, and cause leakage of ions and contents (Kumar et al., 2012). Bioactive metabolites possessing important pharmacological activities are widely distributed in various plant parts, including leaves, flowers, bark, roots, stems, and seeds, and are known to contribute substantially to the treatment and management of numerous diseases and disorders (Bayode et al., 2024). Nonetheless, the specifics of these interactions and mechanisms are intricate; the systematic representation of the present work is shown in **Figure 13**. The lower MIC and MBC values (0.625–5 mg/mL) in this study indicated that the *P. pinnata* leaf extract exhibited strong antibacterial activity against the tested pathogen.

In the acute oral toxicity study, a single dose of *P. pinnata* leaf extract at 2,000 mg/kg body weight was found to be safe in mice, with no mortality or observable effects on food and water intake, body weight, behavior, or overall well-being. While high doses may affect vital organs, gross necropsy revealed no abnormalities in organ size, shape, or weight compared with the control group. These findings indicate that the LD_50_ of the extract exceeds 2,000 mg/kg. According to OECD guideline 423, the absence of mortality suggests that the extract has low toxicity and falls under category 5 (Brondani et al., 2017).

The methanolic extract of *P. pinnata* leaf was evaluated for its antidiarrheal activity using castor-oil-induced models of intestinal motility, fluid accumulation, and defecation frequency. Diarrhea, often resulting from increased intestinal motility and fluid secretion, can be induced by castor oil via its active metabolite, ricinoleic acid, which stimulates peristalsis and enhances mucosal permeability by stimulating prostaglandin biosynthesis (Ammon et al., 1974). This model can reflect both secretory- and motility-related diarrhea. Another mechanism involves the inhibition of Na^+^/K^+^–ATPase activity, which impairs fluid absorption (Yegnanarayan Radha and Shrotri, 1984). Thus, it can be assumed that the extract may reduce electrolyte permeability and suppress prostaglandin-mediated secretion as antidiarrheal activity is likely mediated through both antisecretory and antimotility actions, which are attributed to its bioactive phytochemicals (Viswanatha et al., 2011).

Among the bioactive compounds identified in this study, N-decanoic acid obtained the highest norm%. Higher norm% values suggest that the compound is more abundant in the extract. Factors such as detector response, ionization efficiency, and compound volatility can affect the value. Another compound, neophytadiene, exhibits a range of medicinal properties, including anti-diabetic, analgesic, antipyretic, anti-cancer, anti-inflammatory, antimicrobial, and antioxidant attributes (Swamy, 2017). Similarly, N-decanoic acid and 4-tetradecanol serve as antibacterial and anti-inflammatory agents, respectively (NCBI, 2013). Furthermore, this study documents the antibacterial activity of flutriafol, 1,2,5,6-di-O-isopropylidene-3-O-methane sulfonyl glucofuranose, and spiro{2,4}heptane, 1,2,4,5-tetramethyl-6-methylene, which is found to be at par with the antibiotic ampicillin. These investigations collectively emphasize the abundance of secondary metabolites in *P. pinnata*, which confer remarkable medicinal potential.

Then, using the Swiss Target Prediction database, we predicted and screened for active compounds targeting *P. pinnata*. Using the GeneCards database and a known target, we selected the PBP bacterial target protein. Next, we constructed the PPI network using the STRING database, visual*ized* it with Cytoscape 3.9.1, and selected the highly connected node for further analysis. The network was further subjected to GO and KEGG enrichment analysis to create a pathway–target network. PBPC was the gene most associated with pathways and cell shape. Peptidoglycan synthesis, cell wall biogenesis/degradation, and cell membrane signal pathways were most associated with targets. The network pharmacology analysis suggested that inhibiting PBP receptors disrupts the bacterial cell surface, potentially impairing bacterial growth and cell wall integrity and indirectly reducing the pathogenic burden.

In pharmacological studies, the combination of computational techniques offers a deeper understanding of drug binding mechanisms, affinity, stability, and potential interactions within a biological system. Molecular docking and dynamics can complement experimental findings by providing a detailed mechanistic understanding of ligand–protein interactions. These studies can explain experimental observations, guide further experiments, and suggest modifications to enhance drug efficacy. In the molecular docking study, a total of 22 molecules (see **Table 5**) were selected, and based on a docking score of -8.1 kJ/mol, flutriofol emerged as the best ligand. Other significant compounds include 1,2,5,6-di-O-isopropylidene-3-O-methane sulfonyl glucofuranose and spiro{2,4}heptane, 1,2,4,5-tetramethyl-6-methylene, which demonstrated favorable binding modes with ΔG scores of -6.7 and -6.6 kJ/mol, respectively, compared to the ΔG score of ampicillin, which was observed as -6.3 kJ/mol. Meanwhile, to the best of our knowledge, there are no reports on the binding mechanism of bioactive compounds from *P. pinnata* with the PBP receptor.

In this work, we characterized the bioactive molecules of *P. pinnata* and performed ADME analysis to test the molecules in terms their drug likeness properties. Under ADME, several parameters were analyzed, such as MW, Log P, HBD, HBA, TPSA, GIP, BBBP, number of Lipinski rule violations, and Log Kp, which support the drug likeness properties of the molecules, etc. Most of the parameters showed favorable values for the majority of the molecules, especially with respect to their bioavailability and membrane permeability, consistent with the findings in the study performed by Ralte et al. (2022). Every molecule in this work is thought to have the potential to be a medication, although most of our compounds may be subject to additional investigation as potential therapeutic candidates based on ADME characteristics and molecular property calculations.

This molecular docking study may correlate with a previous study indicating that flutriafol can be an alternative antibacterial and anti-leakage molecule due to its benzene ring (Guo, 2019). In terms of binding interactions, our analysis showed that spiro{2,4}heptane, 1,2,4,5-tetramethyl-6-methylene and 1,2,5,6-di-O-isopropylidene-3-O-methane sulfonyl glucofuranose interacted with PBPs via hydrophobic interactions, in contrast to flutriafol. It was implied that flutriafol, 1,2,5,6-di-O-isopropylidene-3-O-methane sulfonyl glucofuranose, and spiro{2,4}heptane, 1,2,4,5-tetramethyl-6-methylene are potent molecules that might play a crucial role in the inhibitory activity of PBP proteins that control the diarrheal condition. The top three protein–ligand complexes, including PBP in complex with ampicillin, were subjected to MD simulation, and the results were analyzed for structural stability. When comparing the RMSD of PBP proteins in association with flutriafol, 1,2,5,6-di-O-isopropylidene-3-O-methane sulfonyl glucofuranose and spiro{2,4}heptane, 1,2,4,5-tetramethyl-6-methylene, it was found that there was reduced structural dynamics. In contrast to ampicillin, it appeared that the interaction of these ligands with PBPs reduced the conformational flexibility of the PBPs.

In comparison to the binding of spiro{2,4}heptane, 1,2,4,5-tetramethyl-6-methylene to PBPs, there was greater relaxation detected in the analysis of residual fluctuation in PBP proteins in association with flutriafol and 1,2,5,6-di-O-isopropylidene-3-O-methane. The loop area was responsible for the modest changes in residues near 40–50 and 225–250 on the PBP–spiro{2,4}heptane, 1,2,4,5-tetramethyl-6-methylene complex, which were observed in all simulated trajectories. The area of each complex decreased steadily throughout the 100-ns trajectory, as indicated by Rg and SASA analyses. It indicates that fewer amino acid residues are accessible to the environment, suggesting that proteins exhibit some degree of compactness. The results of the PCA indicate a non-periodic conformational shift, whereas complexes with periodic global motion exhibit a considerably more stable cluster. Comparing the H-bond interactions before and after the MD simulation shows that interactions between the receptor’s residues changed throughout the simulation and, at the conclusion of the simulation, remained with the same residues as before. According to molecular docking and MD simulation analyses, flutriafol is the most stable ligand and can bind to PBPs.

A key limitation of this study is the use of crude or partially purified plant extracts, which may lead to misleading or false-positive bioactivities due to the combined effects of secondary metabolites. Thus, bioassay-guided fractionation and purification are crucial to accurately identify and validate the specific bioactive compounds responsible for the antibacterial and antidiarrheal effects. GC–MS primarily detects volatile and thermally stable compounds in crude plant extracts. However, many important plant metabolites, such as polyphenols, flavonoids, glycosides, alkaloids, and other non-volatile constituents, may not be efficiently detected without derivatization, thus potentially overlooking some metabolites. Therefore, LC–MS and HPLC are more suitable for comprehensive bioactive compound profiling, as they better detect, separate, and characterize thermolabile, polar, and high-molecular-weight compounds with higher sensitivity and accuracy.

Another limitation is that the *in vivo* antidiarrheal test mainly used the castor-oil-induced diarrhea model, which might not fully replicate infectious diarrhea caused by enteric pathogens. More comprehensive studies using pathogen-induced models and clinically relevant diarrheagenic bacteria are needed to better mimic infectious diarrhea. Moreover, mechanisms such as antisecretory, antimotility, anti-inflammatory, and intestinal fluid regulation were not thoroughly investigated. The lack of chronic toxicity, pharmacokinetic, and formulation studies limits the application of these findings. Therefore, further detailed pharmacological, toxicological, and clinical research is necessary to develop *P. pinnata* as a reliable and standard*ized* antidiarrheal treatment.

## Conclusion

The outcomes of this study underscore the potent inhibitory activity of *P. pinnata* leaf extract against bacterial pathogens, its antidiarrheal activity in mice, and its antioxidant effectiveness. This robust bioactivity is attributed to the presence of significant bioactive compounds such as neophytadiene, N-deconoic acid, 1,2,5,6-di-O-isopropylidene-3-O-methane sulfonyl glucofuranose, and spiro{2,4}heptane, 1,2,4,5-tetramethyl-6-methylene, which regulate the cell wall and cell membrane biosynthesis by inhibiting PBP activity.

The integration of molecular docking and molecular dynamics simulations elevates our comprehension of drug–protein interactions, binding mechanisms, and the dynamic intricacies of these complexes. As such, the pharmacological exploration of *P. pinnata* holds potential as a resource for managing waterborne bacterial pathogens associated with diarrhea. Future investigations focusing on compound isolation, toxicity profiling, pharmacokinetics, formulation development, and clinical validation are warranted to facilitate the translation of these findings into safe and effective therapeutic applications.

## Data Availability

The original contributions presented in the study are included in the article/supplementary material. Further inquiries can be directed to the corresponding author.
